# Next-Generation Morphometry for pathomics-data mining in histopathology

**DOI:** 10.1038/s41467-023-36173-0

**Published:** 2023-01-28

**Authors:** David L. Hölscher, Nassim Bouteldja, Mehdi Joodaki, Maria L. Russo, Yu-Chia Lan, Alireza Vafaei Sadr, Mingbo Cheng, Vladimir Tesar, Saskia V. Stillfried, Barbara M. Klinkhammer, Jonathan Barratt, Jürgen Floege, Ian S. D. Roberts, Rosanna Coppo, Ivan G. Costa, Roman D. Bülow, Peter Boor

**Affiliations:** 1 Institute of Pathology, RWTH Aachen University Clinic, Aachen, Germany; 2 Institute for Computational Genomics, RWTH Aachen University Clinic, Aachen, Germany; 3 Fondazione Ricerca Molinette, Torino, Italy; 4 Department of Nephrology, 1st Faculty of Medicine and General University Hospital, Charles University, Prague, Czech Republic; 5 John Walls Renal Unit, University Hospital of Leicester National Health Service Trust, Leicester, United Kingdom; 6 Department of Cardiovascular Sciences, University of Leicester, Leicester, United Kingdom; 7 Department of Nephrology and Immunology, RWTH Aachen University Clinic, Aachen, Germany; 8 Department of Cellular Pathology, Oxford University Hospitals National Health Services Foundation Trust, Oxford, United Kingdom; 9 Regina Margherita Children’s University Hospital, Torino, Italy

**Keywords:** Kidney, Biomarkers, Risk factors

## Abstract

Pathology diagnostics relies on the assessment of morphology by trained experts, which remains subjective and qualitative. Here we developed a framework for large-scale histomorphometry (FLASH) performing deep learning-based semantic segmentation and subsequent large-scale extraction of interpretable, quantitative, morphometric features in non-tumour kidney histology. We use two internal and three external, multi-centre cohorts to analyse over 1000 kidney biopsies and nephrectomies. By associating morphometric features with clinical parameters, we confirm previous concepts and reveal unexpected relations. We show that the extracted features are independent predictors of long-term clinical outcomes in IgA-nephropathy. We introduce single-structure morphometric analysis by applying techniques from single-cell transcriptomics, identifying distinct glomerular populations and morphometric phenotypes along a trajectory of disease progression. Our study provides a concept for Next-generation Morphometry (NGM), enabling comprehensive quantitative pathology data mining, i.e., pathomics.

## Abstract (web summary)

Pathology diagnostics still rely on tissue morphology assessment by trained experts. Here, the authors perform deep-learning-based segmentation followed by large-scale feature extraction of histological images, i.e., next-generation morphometry, to enable outcome-relevant and disease-specific pathomics analysis of non-tumor kidney pathology.

## Introduction

Pathology constitutes a cornerstone in the diagnosis and treatment decisions of many diseases. It mainly relies on morphology-based histopathological tissue analysis, which remains manual and requires highly trained expert pathologists. The same is true for nephropathology, a highly specialised area of pathology focusing on the complex diagnostics of kidney diseases. Scoring systems applied by pathologists, such as the Banff-Classification^1^ of kidney transplant pathology or the Oxford classification of IgA nephropathy (IgAN)^2^, have improved standardisation in nephropathology. These scoring systems provide clinically important readouts, e.g., regarding response to therapy or assessing the likelihood of disease progression^2–4^. Despite such scoring systems, pathology diagnostics still remain semi-quantitative, labour-intensive and subjective, sometimes with high inter-observer variability^5,6^.

Progresses in digitisation of pathology enables workflows augmented by advanced image analysis techniques, particularly using deep learning (DL)^7–9^. End-to-end DL algorithms showed encouraging performances in various tasks, mainly explored in oncologic pathology, e.g., in tumour grading^10^, subtyping of cancer variants^11^ and prediction of mutation status^12^. These approaches, although promising, provide only qualitative or semi-quantitative data and their explainability is limited, mostly remaining a “black-box”^13^. An approach to tackle these limitations and enable histopathology data mining is based on extraction of understandable quantitative features of histological structures^14–18^. This however requires precise and effective segmentation of relevant histopathological structures, which can be achieved using DL.

Here, we developed an automated framework for large scale histomorphometry (FLASH) in nephropathology. FLASH extends an existing DL-segmentation model^19^ and is applicable to all morphological injury patterns across major kidney diseases. FLASH-derived quantitative morphometric features could be traced back directly to histology and reflected morphological alterations associated with different diseases, revealed associations of morphological alterations with clinical parameters and provided independent prognostic factors for disease progression in IgAN.

## Results

### Demographic and clinical characteristics of cohort

**Fig. 1:**
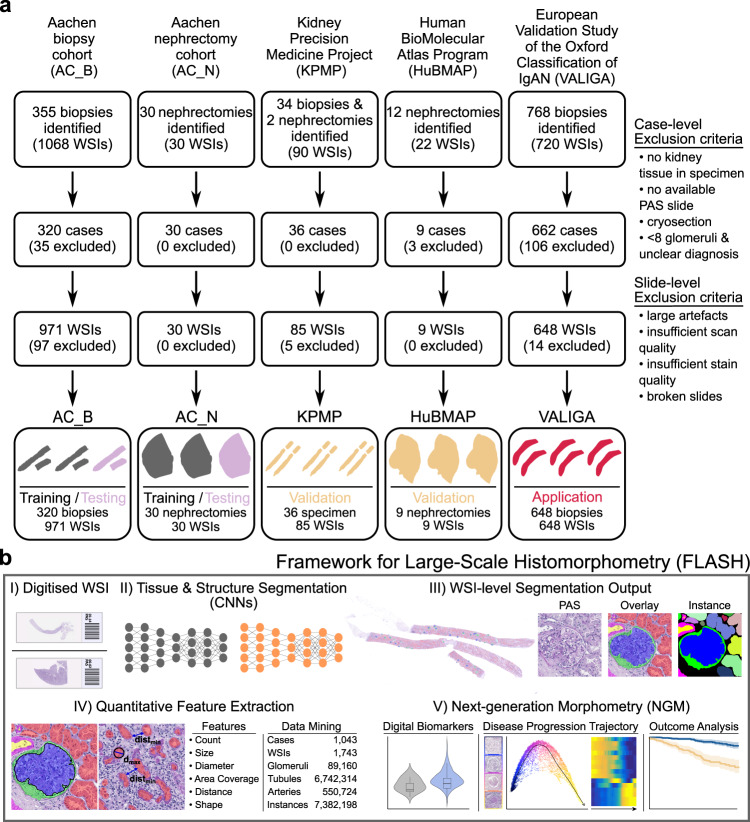
Flowchart of the patient cohorts and the integration of our framework for large-scale histomorphometry (FLASH) into a digital pathology workflow. **a** Overview of the cohort refinement process. Cases and whole-slide images (WSIs) were excluded based on predefined criteria on case- and slide-level. 1043 cases from five cohorts and 1743 WSIs were included in this study. **b** Integration of FLASH into the digital pathology workspace. FLASH combines deep learning-based segmentation with bioinformatics analysis of quantitative morphometric features. The framework consists of two convolutional neural networks (CNNs) for tissue and structure segmentation, computational feature extraction and Next-Generation Morphometry (NGM) analysis. IgAN IgA nephropathy, WSIs whole-slide images, PAS periodic acid schiff.

Two internal, single centre (Aachen Biopsy & Aachen Nephrectomy, AC_B & AC_N), and three external, multi-centre cohorts (HubMAP, KPMP, VALIGA) of kidney biopsies and nephrectomies were included (Fig. 1a). Four cohorts (AC_B, AC_N, HuBMAP, KPMP) covering the whole spectrum of native and transplant pathology were used for development (AC_B & AC_N), testing (AC_B & AC_N) and external validation (KPMP & HuBMAP) of FLASH. The additional VALIGA cohort is a multi-centre international cohort of IgAN patients, i.e., the most common glomerulonephritis worldwide, which was used to analyse the value of FLASH within a potential clinical setting. The two largest cohorts in this study are AC_B and VALIGA, covering approximately 92% of total cases used. Demographic and clinical characteristics between cohorts were comparable, apart from younger patients and more males in the VALIGA cohort, as well as reduced kidney function assessed by estimated glomerular filtration rate (eGFR), which was more common in the AC_B cohort and a higher prevalence of hypertension in the *AC_N* cohort. Patient characteristics of all cohorts are provided in Supp. Table 1.

### Pan-disease segmentation of kidney specimens

**Fig. 2:**
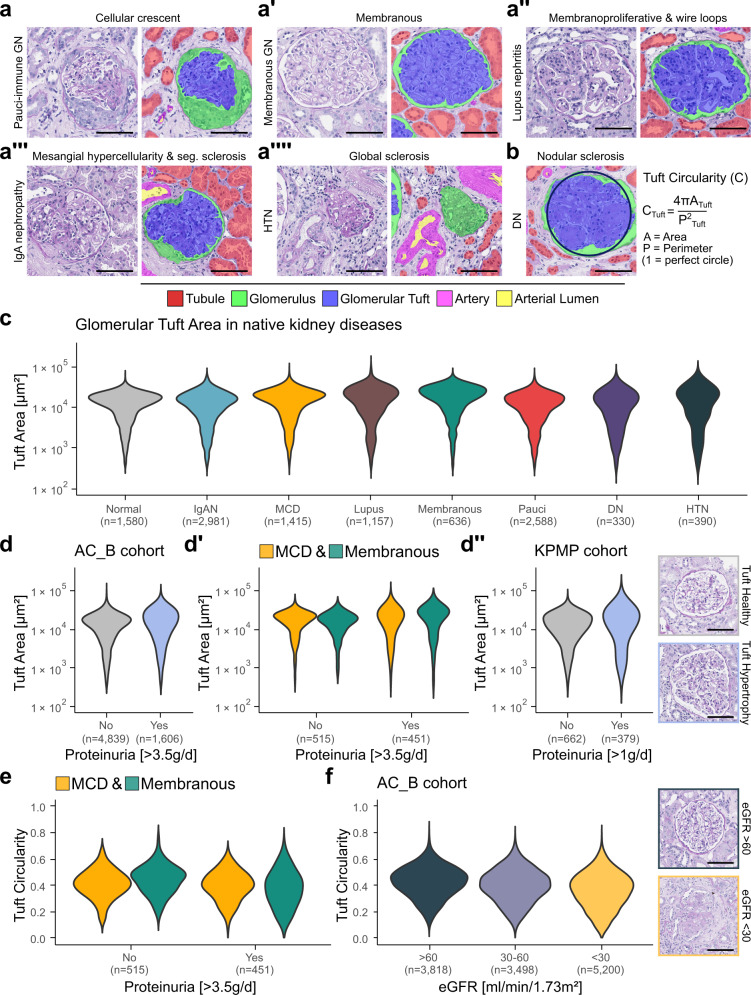
NGM-derived glomerular features reveal distinct morphometric patterns in native kidney diseases and different clinical conditions, such as nephrotic range proteinuria and reduced kidney function. **a**–**a**″″ Segmentation visualisations of glomeruli in major glomerular injury patterns (images stem from the internal *AC_B* cohort excluding training samples). **b** Visual representation for calculation of glomerular tuft circularity as an example of one of the extracted morphometric features. **c** Comparison of glomerular tuft area [μm²] on instance-level with 11,077 instances in different native kidney diseases from the *AC_B* cohort. Glomeruli from biopsies without pathological findings were used as a control (depicted in grey). **d** Feature analysis of glomerular tuft area on instance-level based on nephrotic range proteinuria in all native biopsies from the *AC_B* cohort, **d**′ for glomeruli from biopsies diagnosed with minimal change disease (MCD) or membranous glomerulonephritis (GN) and **d**″ for glomeruli with large proteinuria from the external *KPMP* cohort. Visualisations highlight the increase in glomerular tuft area in cases with nephrotic range proteinuria. **e** Comparison of glomerular tuft circularity on instance-level between cases of MCD and membranous GN with or without nephrotic range proteinuria. **f** Analysis of glomerular tuft shape on instance-level based on reported estimated glomerular filtration rate in all native biopsies from our internal biopsy cohort including additional visualisation examples. Scale bar size is 100 µm. Source data are provided as a Source Data file. GN glomerulonephritis, Seg. segmental, HTN hypertensive nephropathy, DN diabetic nephropathy, IgAN IgA nephropathy, MCD minimal change disease, lupus lupus nephritis, membranous membranous glomerulonephritis, Pauci Pauci-immune glomerulonephritis, eGFR estimated glomerular filtration rate.

**Fig. 3:**
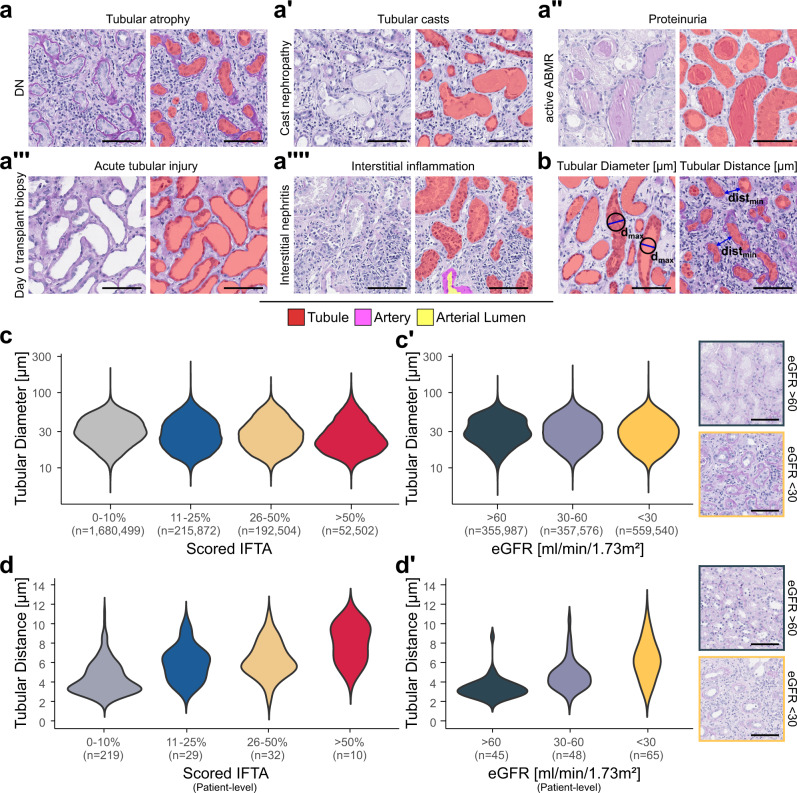
NGM-derived features of tubules and arteries are associated with pathologist derived scoring. **a**–**a**″″ Segmentation visualisations of tubules with large variation in size and shape in various diseases and morphological injury patterns present in the patch. Visualisations stem from the internal *AC_B* cohort excluding training samples. **b** Visual representation of feature calculation of tubular diameter and tubular distance. **c** Feature analysis of tubular diameter on instance-level based on the quantified amount of interstitial fibrosis and tubular atrophy (IFTA) of all biopsies with reported IFTA from the *AC_B* cohort. **c**′ Analysis of tubular diameter on instance-level based on the measured estimated glomerular filtration rate (eGFR) of all native biopsies from our internal biopsy cohort. **d** Feature analysis of tubular distance summarised on patient-level based on the quantified IFTA of all biopsies with reported IFTA from the internal biopsy cohort. **d**′ Analysis of tubular distance summarised on patient-level based on the measured eGFR of all native biopsies from our internal biopsy cohort. Scale bar size is 100 µm. Source data are provided as a Source Data file. DN diabetic nephropathy, ABMR antibody-mediated rejection, *d*_max_ maximum instance diameter, dist_min_ minimum instance distance, IFTA interstitial fibrosis and tubular atrophy, eGFR estimated glomerular filtration rate.

To enable quantitative data mining of kidney histology, the tissue must be precisely separated into relevant histopathological structures, such as glomeruli, tubules, vessels and interstitium. Two streamlined segmentation convolutional neural networks (CNNs) were trained to automate segmentation inference. One CNN was used for kidney tissue segmentation and another for instance-level (e.g., one glomerulus is one instance) structure segmentation of i) glomeruli, ii) their respective tufts, iii) tubules, iv) arteries, v) their respective lumina and vi) non-tissue background (annotation criteria are given in Supp. Table 2). Both segmentation CNNs showed high accuracies in the internal cohorts (AC_B & AC_N) assessed by Dice-similarity-coefficients (on class- and instance-level), F1-score and positive predictive value (Table 1, Supp. Fig. 1). The structure segmentation CNN correctly segmented glomeruli and glomerular tufts across all injury patterns, even in complex cases such as crescents, segmental sclerosis or a membranoproliferative pattern (Fig. 2a). High accuracy was also observed for tubules despite large variations in size and shape, e.g., in tubular atrophy or light chain casts (Fig. 3a). Arteries and especially their lumina were segmented with lower precision (Table 1). Despite large differences in staining protocols (Supp. Fig. 2), segmentation accuracy was comparable or even better in the external, multi-centre cohorts used for validation only (HuBMAP & KPMP), indicating broad generalisability (Table 1 and Supp. Fig. 3). Small segmentation errors were detected in all classes (Supp. Fig. 4). Over 2000 additional examples of segmented structures can be found at git-ce.rwth-aachen.de/labooratory-ai/flash/-/tree/main/exemplary_CNN_segmentations.

**Table 1:** Performance metrics for the tissue segmentation convolutional neural network (CNN) and structure segmentation CNN

Tissue segmentation CNN												
	AC_B			AC_N			KPMP			HuBMAP		
Class	DSC											
Kidney Tissue	0.99			0.98			0.92			0.99		

Structure segmentation CNN												
	AC_B			AC_N			KPMP			HuBMAP		
Class	iDSC	F1	PPV	iDSC	F1	PPV	iDSC	F1	PPV	iDSC	F1	PPV
Tubule	0.89	0.93	0.94	0.87	0.92	0.91	0.91	0.94	0.95	0.89	0.94	0.95
Glomerulus	0.93	0.97	0.99	0.91	0.92	0.95	0.94	0.97	0.98	0.92	0.95	0.94
Glomerular tuft	0.87	0.91	0.90	0.91	0.95	0.95	0.94	0.98	0.98	0.94	0.98	0.99
Non-tissue background	0.94	0.96	0.96	0.80	0.84	0.80	0.93	0.96	0.97	0.90	0.92	0.93
Artery	0.73	0.77	0.84	0.64	0.69	0.77	0.64	0.66	0.69	0.70	0.70	0.78
Arterial lumen	0.72	0.78	0.87	0.52	0.56	0.59	0.59	0.63	0.75	0.71	0.78	0.86

*iDSC* instance Dice-similarity-coefficient measuring the maximum overlapping area in pixels for each instance between model prediction and ground truth, *F1* F1-Score, *PPV* positive predictive value.

Segmentation performance of the tissue segmentation CNN was evaluated by calculating Dice-similarity-coefficients (DSCs). Segmentation performance of the structure segmentation CNN was evaluated by averaging all calculated metrics from each instance in all test/external validation images.

Taken together, FLASH allowed a broad, “pan-disease” applicability across all common diseases and morphological injury patterns in multi-centre kidney datasets.

### Glomerular morphometry is associated with specific diseases and clinical readouts

Applying FLASH enabled the extraction of features of more than 11,000 glomeruli and glomerular tufts in the *AC_B* cohort, the subsequent large-scale comparisons of glomerular morphometric features (Supp. Table 3) and their distributions in common native kidney diseases (Fig. 2c and Supp. Fig. 5A, B). The median glomerular tuft area was larger by 19.71% (95% CI [10.65, 28.83%]) in lupus nephritis, 18.9% (95% CI [12.42, 25.91%]) in minimal change disease (MCD) and 40.54% (95% CI [30.99, 51.5%]) in membranous glomerulonephritis (GN) all with increased interquartile range (IQR) and significant changes in tuft area distribution (all adjusted (adj.) *p* < 0.01) compared to the normal baseline (Fig. 2c and Supp. Table 4). This effect could also be observed for full glomeruli (i.e., tuft + Bowman’s space + capsule) in lupus nephritis (7.41% increase, 95% CI [0.77, 15.94%]), MCD (7.91% increase, 95% CI [0.33, 15.5%]) and membranous GN (25.21% increase, 95% CI [15.14, 34.92%]) and their respective distributions (all adj. *p* < 0.01). The change of the full glomerular area was less prominent than that of the tuft (Supp. Fig. 5A and Supp. Table 4). In diabetic (DN) or hypertensive nephropathy (HTN) distributions of glomerular and tuft areas were more variable with larger IQRs. This was especially pronounced in HTN biopsies where the median glomerular tuft area was larger compared to the normal baseline, while the percentage of glomeruli without a tuft (e.g., globally sclerotic, or empty Bowman’s space) was considerably higher as well (45.93% compared to 19.68%).

Next, the hypothesis that proteinuria is associated with glomerular hypertrophy was investigated^20^. The glomerular tuft areas in native *AC_B* cases with vs. without nephrotic range proteinuria (i.e., > vs. <3.5 g/d) were larger (9.71%, 95% CI [2.81, 15.81%], Fig. 2d and Supp. Table 5) with significant changes in their distribution (*p* < 0.01). Analysis of diseases typically associated with proteinuria, i.e., MCD and membranous GN confirmed these findings with significantly different distributions (both *p* < 0.01) and an average increase of mean tuft area by 10.69% (95% CI [1.1, 20.81%]) in MCD and median tuft area by 51.01% (95% CI [34.3, 77.62%]) in membranous GN (Fig. 2d′ and Supp. Table 5). Interestingly, the median tuft area was slightly smaller in MCD. A similar increase in glomerular tuft area by 18.7% (95% CI [−3.17, 35.6%]) was found in the KPMP cohort (Fig. 2d″ and Supp. Table 5). While the distributions between the two groups in the KPMP cohort were significantly different (*p* < 0.01), we could only observe a trend in the glomerular tuft area increase. Tuft circularity in MCD did not significantly change in nephrotic range proteinuria (2.5% decrease, 95% CI [−2.44, 7.32%], *p* = 0.39). In contrast, distribution of tuft circularity changed significantly (*p* < 0.01) and median tuft circularity decreased by 19.57% (95% CI [11.96, 28.26%]) in membranous GN with nephrotic range proteinuria (Fig. 2e and Supp. Table 5).

In all native kidney biopsy cases from the *AC_B* cohort, the tuft circularity progressively decreased with kidney function loss (13.95% overall decrease, 95% CI [11.63, 16.28%], in cases with eGFR >60 to eGFR of 30-60 (6.98% decrease, 95% CI [4.65, 9.3%]) to eGFR <30 ml/min/1.73 m² (7.5% decrease, 95% CI [5.0, 10.0%], Fig. 2f and Supp. Table 6) resulting in significantly different distributions between groups (all adj. *p* < 0.01). Furthermore, the tuft circularity decreased by 24.44% (95% CI [17.78, 26.67%]) in DN, 18.89% (95% CI [13.33, 24.44%]) in HTN and 20.0% (95% CI [17.78, 22.22%]) in pauci-immune GN with significant changes in their respective distributions (all adj. *p* < 0.01) compared to normal biopsies (Supp. Fig. 5B and Supp. Table 4).

Taken together, FLASH allowed large scale quantitative analysis of glomerular morphometry, revealing clinico-morphological associations.

### Morphometry of the tubulointerstitium and vasculature is linked to kidney function & hypertension

Since the tubulointerstitium and vasculature are often damaged in kidney diseases, FLASH was used to extract features of over two million tubular instances. These were compared based on the reported diagnosis, histopathological scoring and kidney function estimated by eGFR.

The tubular diameter decreased in DN (by 16.04%, 95% CI [15.39, 16.63%]), HTN (by 14.81%, 95% CI [13.77, 15.17%]) and IgAN (by 4.58%, 95% CI [3.96, 5.03%]) with significant changes in tubular diameter distribution (all adj. *p* < 0.01) compared to normal biopsies (Supp. Fig. 5C and Supp. Table 4). When grouping cases based on the interstitial fibrosis and tubular atrophy score (IFTA) taken from the pathology reports, the tubular diameters continuously decreased from none/marginal (0-10% IFTA) to mild (11-25% IFTA) to moderate (26-50% IFTA) to severe (>50% IFTA) and distributions of tubular diameters were significantly different compared to none/marginal IFTA (all adj. *p* < 0.01, Fig. 3c and Supp. Table 7). Conversely, the tubular distance increased in biopsies with mild, moderate and severe IFTA compared to none/marginal IFTA (Fig. 3d and Supp. Table 7). Similar changes in the distribution of tubular morphometry were observed when cases were grouped based on stratified eGFR. The tubular diameter progressively decreased with kidney function loss (5.84% overall decrease, 95% CI [5.64, 6.24%]), in cases with eGFR >60 to eGFR of 30–60 (2.63% decrease, 95% CI [2.49, 2.99%]) to eGFR <30 ml/min/1.73 m² (3.3% decrease, 95% CI [3.03, 3.54%], Fig. 3c′ and Supp. Table 6) while the tubular distance increased (86.42% overall increase, 95% CI [58.36, 96.99%]) in cases with eGFR >60 to eGFR of 30-60 (39.81% increase, 95% CI [26.28, 61.66%]) to eGFR <30 ml/min/1.73 m² (33.33% increase, 95% CI [8.89, 43.34%], Fig. 3d′ and Supp. Table 6).

Arteriosclerosis is a common chronic vascular injury pattern in kidney diseases, currently only reported in gross grades. The distributions of artery wall diameter (i.e., wall thickness, Supp. Fig. 6C) and artery lumen diameter were significantly different in cases with none to moderate to severe reported arteriosclerosis (all adj. *p* < 0.01). While the measured wall diameters increased, the lumen diameters steadily decreased with severity of arteriosclerosis (Supp. Fig. 6 and Supp. Table 8). The median wall diameters of arteries and arterioles were larger by 8.59% (95% CI [5.67, 11.35%]) in the *AC_B*, 10.77% (95% CI [7.43, 15.3%]) in the *AC_N* and 20.33% (95% CI [13.37, 27.13%]) in the HuBMAP cohort based on the presence of hypertension (Supp. Fig. 6D and Supp. Table 8). Distributions of wall diameter significantly changed in the AC_B, AC_N and HuBMAP cohorts (all *p* < 0.01) based on the presence of hypertension while in the KPMP cohort the distributions did not show significant differences (*p* = 0.15) and only an increase of median wall diameter by 2.4% was observed (95% CI [−19.34, 11.55]). Diameters of the arterial lumen decreased in all four cohorts (Supp. Table 8). Taken together, vascular features reflect the pathologist’s assessment of arteriosclerosis, are associated with the presence of hypertension, and allow quantitative assessment of vascular alterations.

### Morphometric features are predictive of disease progression in IgA nephropathy

**Fig. 4:**
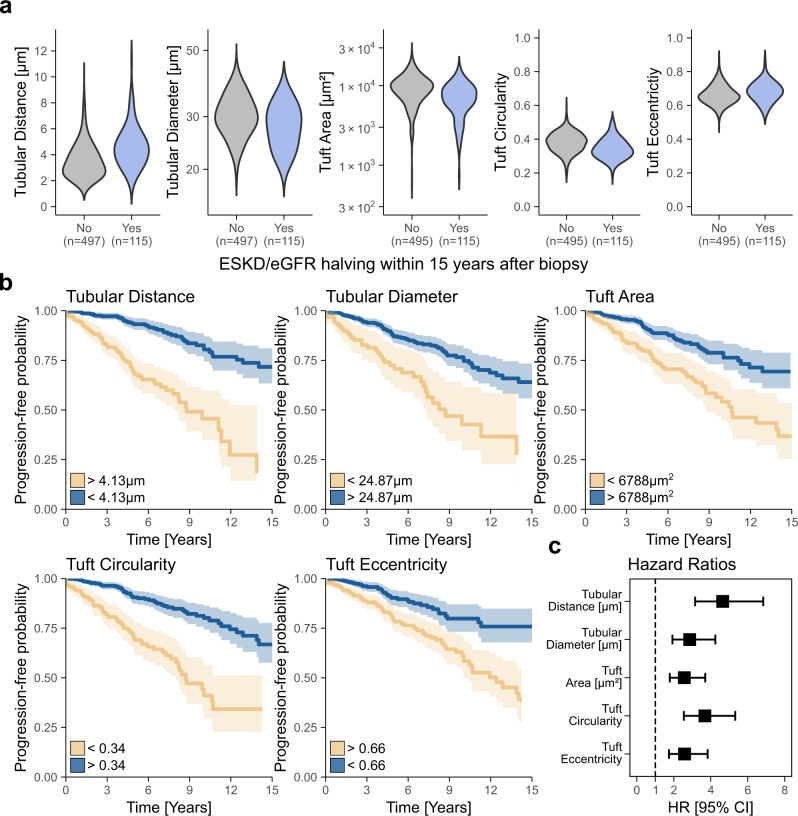
NGM-derived quantitative features are predictive of disease progression in IgA nephropathy (IgAN). **a** Comparison of five predictive digital biomarkers summarised at patient-level based on reaching the defined composite endpoint, i.e., end-stage kidney disease and/or halving of initial estimated glomerular filtration rate (eGFR) within 15 years after biopsy. **b** Univariate Cox proportional hazards models for 644 patients of the five predictive features summarised at patient-level including 95% confidence intervals. In three cases no glomerular tuft was segmented, and no shape features were calculated. Cumulative events for each group in the univariate Cox proportional hazard models are provided in Supp. Table 9. **c** Hazard ratios (centre) and their 95% confidence interval (error bars) from the univariate Cox proportional hazard models of the respective features. Source data are provided as a Source Data file. ESKD end-stage kidney disease, eGFR estimated glomerular filtration rate, HR hazard ratio, CI confidence interval.

To assess the utility of FLASH for outcome prediction in a clinical setting, the multi-centre VALIGA cohort of IgAN patients was analysed. Disease progression was defined as reaching the composite endpoint of end-stage kidney disease (ESKD) and/or halving of the initial eGFR assessed at the time of biopsy within fifteen years after biopsy. Median follow-up time was 4.72 (IQR: 5.28) years. 17.86% of patients (*n* = 115) reached the composite endpoint (13.04% due to ESKD, 26.09% due to eGFR halving and 60.87% due to both endpoints) within a median time of 4.53 (IQR: 5.17) years. Comparison of biopsies of patients reaching the composite endpoint vs. those who did not, revealed a decrease in tuft circularity (by 12.87%, 95% CI [10.08, 17.15%]), tuft area (by 26.91%, 95% CI [20.12, 41.52%]), tubular diameter (by 5.51%, 95% CI [1.32, 6.97%]), and an increase in tubular distance (by 35.9%, 95% CI [25.94, 46.73%]) and tuft eccentricity (by 4.13%, 95% CI [2.48, 5.96%]) with significant changes in respective feature distributions (*p* < 0.01) (Fig. 4a). Univariate Cox proportional hazards models for these five features showed that patients with certain feature expressions at the time of biopsy displayed a faster decline of disease progression-free probability and a higher risk of reaching the composite endpoint (Fig. 4b, c). Adjusted multivariate analysis for each predictive feature as well as age, sex, MEST-C score and eGFR at the time of biopsy confirmed tubular distance (HR 2.03, 95% CI [1.24–3.32], *p* < 0.01), tubular diameter (HR 1.73, 95% CI [1.12–2.68], *p* < 0.05), tuft area (HR 1.51, 95% CI [1.02–2.25], *p* < 0.05), tuft circularity (HR 2.04, 95% CI [1.38–3.02], *p* < 0.01) and tuft eccentricity (HR 1.73, 95% CI [1.15–2.61], *p* < 0.01) as independent predictors for reaching the composite endpoint, being significantly associated with disease progression (Supp. Table 9–12). To further compare the digitally derived morphometric biomarkers with traditional histopathology scoring for IgAN, two Cox proportional hazards models were fitted, i) Digital Biomarkers (including all five digital features, age, sex and initial eGFR) and ii) MEST-C (including M, E, S, T, C, age, sex and initial eGFR; Supp. Table 11). The fitted Digital Biomarkers model (C-statistic =0.79 ± 0.03, AIC = 1195, BIC = 1217) was equivalent to the *MEST-C* model (C-statistic = 0.79 ± 0.02, AIC = 1204, BIC = 1226). Combining the Digital Biomarkers and MEST-C model into a third, hybrid model resulted in a slight improvement (C-statistic=0.82 ± 0.02, AIC = 1189, BIC = 1224).

### Morphometric phenotypes along a disease progression trajectory

**Fig. 5:**
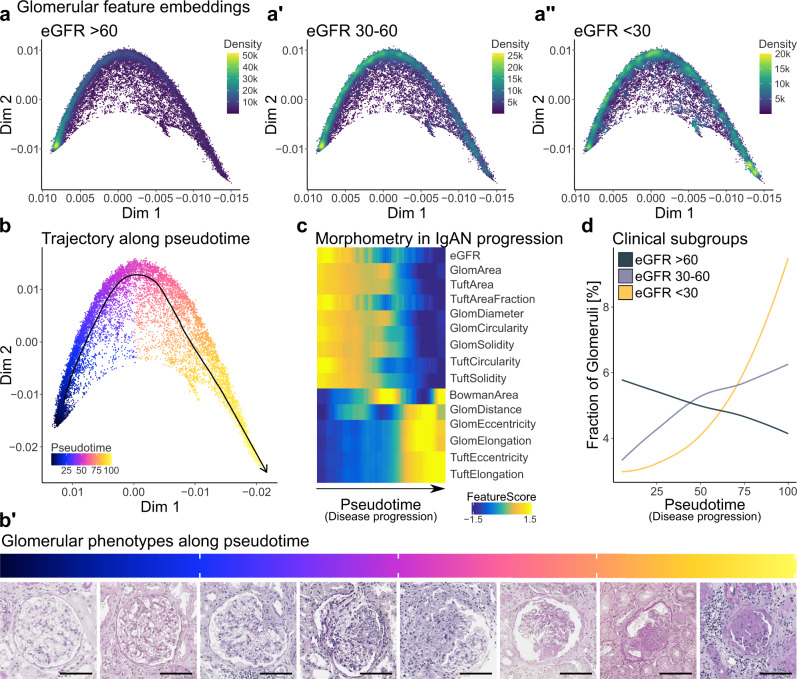
Pseudotime analysis of NGM-derived glomerular features identifies distinct glomerular groups along a trajectory of disease progression in IgA nephropathy (IgAN). **a**–**a**″ Diffusion map embedding of 24,227 glomerular instances with 14 morphometric features with IgAN based on the reported estimated glomerular filtration rate (eGFR) [ml/min/1.73 m²]. **b** Diffusion map of glomerular instances with pseudotime indicating ordering of glomerular instances along their progression from healthy to diseased. **b**′ Visualisation of glomerular phenotypes along the pseudotime. **c** Scaled feature expression heatmap including eGFR along the pseudotime trajectory. **d** Morphometric progression of glomerular instances in clinical subgroups based on the overall reported eGFR. Scale bar size is 100 µm. Source data are provided as a Source Data file. eGFR estimated glomerular filtration rate, Dim diffusion map, IgAN IgA nephropathy.

Animal models and experience from kidney biopsy diagnostics allow generating hypotheses on the course of morphological alterations during disease progression, however, an approach to quantitatively analyse this was missing. To tackle this, an unsupervised analysis using diffusion maps was performed to find major axes of glomerular morphometric changes in IgAN, revealing clusters of glomerular instances attributed to the overall kidney function measured by eGFR (Fig. 5a–a″). Based on this, a trajectory and an estimated pseudotime score were determined, where glomeruli progress from a healthy to a diseased morphometric phenotype (Fig. 5b). Histologic examples of glomeruli along the pseudotime supported morphological changes progressing from normal to increasingly diseased phenotypes with higher pseudotime scores, e.g., with increasing mesangial expansion and sclerosis (Fig. 5b′ and Supp. Fig. 7).

A feature expression heatmap along the pseudotime revealed glomerular morphometric alterations associated with IgAN disease progression, e.g., decreasing tuft area and tuft circularity, and increasing tuft eccentricity and elongation, resulting in smaller and more deformed glomerular tufts (Fig. 5c). The fraction of glomerular instances at the beginning of the pseudotime trajectory that belong to patients with preserved kidney function (>60 ml/min/1.73 m²) continuously decreased along the pseudotime (Fig. 5d). On the other hand, the fraction of glomeruli from patients with considerably reduced kidney function (<30 ml/min/1.73 m²) constantly increased along the pseudotime trajectory, indicating that the pseudotime represents the disease progression of IgAN towards ESKD in glomerular populations (Fig. 5d). A similar trajectory from healthy to disease along the pseudotime could be observed using patient level aggregated glomerular and tubular features in the VALIG*A* trial (Supp. Fig. 9).

Automated visualisation of image patches enabled displaying morphometric outliers of glomeruli and tubules in a single, representative case of IgAN from the AC_B cohort. Morphometric outliers of structures of interest were displaying various pathological lesions i.e., crescents, segmental sclerosis or tubular atrophy (Supp. Fig. 9) which could enable fast-track assessment of kidney histopathology.

## Discussion

Our study presents a proof-of-concept for large-scale automated extraction of large-scale quantitative morphometric data from histopathology, i.e., Next-Generation Morphometry (NGM). For this, a deep learning-based instance segmentation and quantification framework (FLASH) was implemented. FLASH was developed and validated in heterogeneous multi-centre datasets using both kidney biopsies and nephrectomies and a large variety of diseases. The segmentation accuracy of FLASH was high across cohorts, indicating broad generalisability. We focused on nephropathology, since the kidney is one of the most complex organs in pathology diagnostics, requiring a high level of specialisation, and representing a challenging use case. NGM provides the basis for histopathology morphometry, an “omics” approach we propose to term “pathomics”.

Omics technologies comprehensively quantify biomolecules in an automated manner and on a large scale, e.g., DNA in genomics, RNA in transcriptomics and proteins in proteomics^21^. These approaches are increasingly performed in a comprehensive, multi-omics fashion^22^ and with spatial resolution^23,24^. Although morphological alterations in diseases are very well recognised and have been used for diagnostics for over a century, approaches for omics-based analysis of histopathology were missing. NGM and pathomics could serve as a complementary approach to the molecular omics techniques, providing objective, tissue-based, quantitative (geometrical) information on histological structures. Compared to established omics techniques, which are continuously improved, NGM is currently in its infancy. It is expected that NGM will undergo prominent development, particularly given that the technological prerequisites are largely met, i.e., the instruments for high-throughput digitisation of histology slides, graphics processing units (GPUs) and storage are increasingly available and affordable. E.g., in this study we were able to analyse 7,382,198 instances of histological structures with 6,742,314 tubules, 89,160 glomeruli and 550,724 arteries, showing that NGM can be used to provide data on histology at an unprecedented scale.

Similar to Next-generation Sequencing and genomics, which have revolutionised research and diagnostics by comprehensive genetic molecular characterisation, NGM opens new frontiers in quantitative assessment of morphology. As a first proof-of-concept we have shown the potential utility of NGM and pathomics for quantitative kidney histopathology data mining, providing clinically relevant and complementary readouts that can constitute an important step towards precision medicine.

Patients with MCD or membranous GN and nephrotic range proteinuria showed a prominent increase in mean glomerular tuft area, compared to those without. In MCD-patients, larger glomeruli identified by manual analysis were previously associated with an increased risk for kidney function deterioration and development of glomerular sclerosis^25^. With FLASH and NGM, such morphological biomarkers can now be assessed automatically across all diseases. Importantly, FLASH revealed a decrease in tuft circularity in membranous GN patients with nephrotic range proteinuria, but not in MCD, indicating different mechanisms of glomerular hypertrophy in these diseases. The tuft circularity progressively decreased across all native diseases in patients with decreased kidney function, indicating that this might be a general feature of kidney function decline. Thereby, NGM can provide novel findings and generate novel research questions based on morphology.

NGM and FLASH enabled the identification of morphological features that are independent predictors of kidney function decline in IgAN, such as the smallest distance between tubular instances, the glomerular tuft circularity, the glomerular tuft eccentricity or the tubular diameter. While some of these were expected, and confirmed previous concepts, e.g., the distance between tubules reflecting interstitial fibrosis, others, such as tuft circularity and tuft eccentricity, were unexpected. These features could be used as a set of digital biomarkers, potentially improving the predictive value and reproducibility of histopathology diagnostics. Accordingly, a combined model of only a few of these digital biomarkers proved to be equivalent compared to a validated standard histopathological scoring system, i.e., the MEST-C score^26^. The advantage of using NGM over a pathologist-derived score is that it is quantitative and fully automated. Therefore, we expect that NGM is better reproducible, more precise, faster and spares the time of pathologists.

Adapting techniques designed to analyse other omics data, such as single cell sequencing data, we identified a trajectory of disease progression in a low dimensional embedding of glomerular features in IgAN. This allowed a granular analysis of progression of glomerular phenotypes from healthy to diseased, which can be seen as an unsupervised way of identifying histologic features relevant for disease progression. This first proof of concept shows that NGM-based data can be used to make histopathology analysis quantitative, capturing more subtle changes which better reflect the biological reality of disease progression and modelling disease progression relevant pathologies.

Some studies previously described the potential of morphometric analysis of histology^19,27–32^. Although very specific, these scoring systems were applicable only in specific use cases and particular pathological alterations. NGM and FLASH follow a holistic approach of morphometric analysis, prioritising automated data mining, thus enabling a wider variety of possible downstream analyses, i.e., an exploratory approach comparable to other omics techniques.

Currently, a major focus in computational pathology is the development of end-to-end DL solutions, which mostly provide qualitative results, e.g., a disease class or mutational status^12,33–35^. On the contrary, NGM and FLASH use segmentation as a basis for subsequent large-scale quantitative data mining. Compared to end-to-end pipelines, NGM provides an alternative approach with several advantages. The results are visually verifiable, can be easily checked by pathologists, and are therefore interpretable. This is often not the case in end-to-end DL solutions, which remain a black-box in terms of explainability. Therefore, quantitative histology features remain comprehensible, even if clustered in a lower dimensional space. This can help reduce potential scepticism towards DL based systems that might hinder clinical application.

In nephropathology, most diseases, including IgAN, are rare diseases. Large data repositories allowing the effective and robust development of end-to-end DL pipelines in nephropathology are missing, making the development of such pipelines considerably more difficult compared to oncological pathology. Only very few nephropathology datasets with clinical information are publicly available, the largest being KPMP and HuBMAP. We have included these in our study, yet their WSIs represented only ~8.5% of the whole dataset. Additionally, the format of the clinical data differed, not allowing to combine all cohorts in all analyses. Current initiatives, such as the BIGPICTURE project^36^, or grand challenges^37^ might help to tackle this in the future. We openly provide our pathomics datasets for KPMP and HuBMAP, enriching these publicly available cohorts with complementary pathomics data. In comparison, NGM and FLASH do not require large datasets for development and can be applied to any type of disease, including rare diseases such as in MCD, IgAN, etc.

This study has several important limitations. Currently, FLASH only includes a few, easily explainable morphometric features, as we focused on providing a proof-of-concept of the utility of NGM. We have analysed these features in formalin-fixed and paraffin-embedded (FFPE) material; therefore, they could potentially reflect changes from fixation, which is an inherent limitation of histology-based studies. Future research could investigate potential differences in the morphometry of samples with different fixations. One of the challenges we encountered was the large variability within the stainings. The precision of segmentation was consistently high despite this variability, resulting in robust morphometric data. However, the colour variability prohibited us from extracting additional, e.g., colour or texture-based features. Further developments should focus on colour normalisation approaches, a larger number of additional morphological features, and inclusion of subvisual features such as texture to provide even more comprehensive morphometric data.

Another limitation is that FLASH is not generic for any kind of tissue, but specifically developed for the kidney. Particularly because of the required tissue-specific segmentation and different stains used in various organ histopathology analysis, it is currently unlikely to have a pipeline applicable for every kind of tissue histology. In addition, generating the ground truth for the segmentation algorithm requires considerable effort and time-investment by expert pathologists, which is a limitation in comparison to end-to-end approaches, which can be trained in a weakly supervised way with very little manual overhead. While segmentation accuracy was consistently high for other compartments, arteries and their lumina were difficult to segment, resulting in lower performance. This might be mitigated by performing many more expert annotations. In another kidney segmentation study, almost 20,000 annotations were necessary to allow segmentation of peritubular capillaries in mostly normal appearing tubulointerstitium of MCD patients^38^. In addition, the data on severity of arterial hypertension were not available, making the analyses of arterial feature distributions rather preliminary. We present data on multiple disease entities. However, a sufficient number of patients and outcome data were only available for IgA-Nephropathy in the VALIGA trial, limiting outcome analyses and adjustments for potential confounders to this cohort and disease entity. Future studies should investigate larger cohorts to validate the differences found in feature distributions in other diseases. Furthermore, the here used cohorts mainly included patients with European and in part Asian ancestry, and anthropometric data were largely not available. Another limitation is the retrospective design of this study. However, this study should serve as the basis for designing potential future prospective trials investigating the predictive potential of NGM.

In conclusion, our study lays the groundwork for introducing NGM and pathomics for explainable, quantitative, histopathology analysis and pathomics.

## Methods

### Ethics statement

Data collection and analysis in this study was performed in accordance with the Declaration of Helsinki and was approved by the local ethics committee of the RWTH Aachen University (EK-No. 315/19). All analyses were performed retrospectively in an anonymous fashion and the need for informed consent was waived by the local ethics and privacy committee for all datasets.

### Cohort assembly and sample collection

For development, validation and application of FLASH, whole-slide images (WSIs) and clinical data from five cohorts were gathered: two internal, i.e. in-house cohorts from the Institute of Pathology in Aachen, for development, i.e. Aachen Biopsy (AC_B) and Nephrectomy (AC_N), and three external cohorts from other centres, two of which were used for validation, i.e. Kidney Precision Medicine Project (KPMP, NCT04334707)^39^ and the Human BioMolecular Atlas Program (HuBMAP)^40^, and the third, the VALIGA trial, for a disease-specific application use case (Fig. 1a). Following exclusion criteria were used in all cohorts: (i) no kidney tissue in the specimen, (ii) no Periodic Acid Schiff (PAS)-slide available, (iii) only cryosections available and (iv) containing less than eight glomeruli, unless a definitive pathological diagnosis could be made, (v) large artefacts present on the slide, (vi) insufficient scan quality (e.g., major part of tissue being out of focus and blurred), (vii) insufficient stain quality (e.g., unstained tissue) and (viii) broken slides (Fig. 1a).

### Development cohorts

Aachen Biopsy cohort (AC_B). A database search identified 355 kidney biopsy cases in the archive of the Institute of Pathology of the RWTH Aachen university clinic within the inclusion period (January 1st 2017—May 1st 2021). Biopsies were either native kidney or indication or protocol transplant biopsies. Diagnoses for all cases are given in Supp. Table 13.

In the Aachen Nephrectomy cohort (AC_N) 30 nephrectomy specimens (inclusion period: 2013 − 2021) were included, appreciating that nephropathology is not limited to biopsy specimens and aiming at applicability also in nephrectomy samples. The AC_N cohort consists of 13 transplant nephrectomies due to severe complications and 17 tumour nephrectomies, including only non-tumour tissue away from tumour borders. Both groups reflect a broad morphological spectrum of histopathology. More tumour nephrectomies than transplant nephrectomies were included since they are more common in routine diagnostics.

For all cases from the AC_B & AC_N cohorts the diagnosis, histopathological scores and clinical data were collected when available. Final diagnoses and histopathological scores were gathered from the pathology reports. Histopathological scoring and diagnosis were based on the consensus of at least two trained nephropathologists. The following information on histological structures were collected from the reports when available: number of glomeruli, number of globally sclerotic glomeruli, information regarding the presence and severity of arteriosclerosis, and the extent of interstitial fibrosis and tubular atrophy (IFTA). Furthermore, clinical data including laboratory findings and ICD-10 coded diseases were collected if available within the pathology laboratory information system (Supp. Table 13). Estimated glomerular filtration rate (eGFR) derived by using the Chronic Kidney Disease Epidemiology Collaboration (CKD-EPI) Equation^41^ [ml/min/1.73 m²] and proteinuria [mg/d], were gathered if available at the time of biopsy. Presence of hypertension regardless of aetiology was assessed based on ICD-10 codes. Non-availability of clinical or pathological data did not automatically lead to exclusion (please see exclusion criteria).

Before digitalisation of slides all patients were anonymised and given a unique patient identifier. Overall, 320 biopsy cases from 297 patients (number of biopsies per patient—mean: 1.08, min: 1, max: 4) were included in the AC_B cohort. There was a mean of 3.03 Periodic Acid Schiff (PAS)-stained whole-slide images (WSIs) per biopsy case (min: 1, max: 7).

In both cohorts 1–3 µm thick formalin-fixed and paraffin-embedded sections were used. All slides from the AC_B, AC_N and VALIGA cohorts were digitised using an Aperio AT2 whole-slide scanner with a 40x objective (Leica Biosystems, Wetzlar, Germany).

### External validation cohorts

Two external publicly available cohorts from independent consortia were included to validate the generalisability of our CNNs. The cohort from the KPMP (accessed on 15th March 2021) consists of 90 PAS-stained WSIs from patients with either acute kidney injury (AKI), chronic kidney disease (CKD) or healthy tumour nephrectomies. It included 34 biopsy and two nephrectomy cases. After the exclusion process, 85 PAS-stained WSIs were included in the analysis. The cohort from HubMAP contains 22 nephrectomy specimens from 12 deceased organ donors. In all, 13 cryo-sections were excluded since they were out of distribution (we only trained on FFPE material), with the final cohort consisting of nine nephrectomy WSIs from nine cases. Additionally, clinical data from both cohorts were gathered when available (Supp. Table 13).

### Specific application cohort

After development was finished, the FLASH architecture was applied to the multi-centre *VALIGA* trial which represents one of the largest biopsy cohorts of patients with IgAN. From the initial cohort, 768 cases could be identified and digitised (scanned). The following problems prevented more cases from being included: (a) it was not possible to identify the slide label (e.g., because it faded, or fell off), or (b) slides broke during transport. Overall, 106 cases were excluded, since they met at least one of the above-mentioned exclusion criteria, most often not available digital PAS-stained slides. An additional 14 cases were excluded on slide level due to artefacts, with in total, 648 PAS-stained WSIs of 648 cases being included (Fig. 1a).

### Framework development

FLASH consists of an automated three-step approach: (i) a CNN that automatically segments kidney tissue on a WSI discarding all non-kidney tissues (e.g., adipose or muscle tissue), (ii) another CNN that segments histological structures of the kidney tissue segmented by the first CNN and (iii) hand-crafted feature extraction for segmented structures (Fig. 1b). The framework is applicable to the whole morphological spectrum of non-neoplastic kidney diseases.

### Generation of annotations

For the kidney tissue segmentation, we annotated kidney tissue on slide-level. For the segmentation of histological structures, we annotated patches of size 174 × 174 µm² using the following six classes (Supp. Table 2): (1) full glomerulus (including the tuft), (2) glomerular tuft, (3) tubule, (4) artery (including lumen, intima and media), (5) arterial lumen and (6) non-tissue background (including veins with a diameter of >30 µm). We focused on these classes since they represent the major kidney compartments and can be reproducibly annotated even in severe diseases. Overall, we annotated 1056 WSIs for the tissue segmentation and 4031 patches and 27,287 structures for the structure segmentation in the four development and validation cohorts (Supp. Tables 15–16). Ground truth annotations of histological kidney structures were performed by an experienced nephropathologist and two trained medical students using QuPath^42^, which is the most widely used open-source software for computational pathology applications. In a second step the nephropathologist corrected all annotations. Annotations of difficult/questionable structures were discussed in regular meetings with another nephropathologist. All annotators were instructed to follow a standard operating procedure with clear cut definitions for all six classes (Supp. Table 2). Besides, the following procedure was performed to accelerate the time-consuming manual annotation process: First, a quarter of the training dataset was annotated and used to train a prior segmentation model. Then additional patches were selected for annotation, focusing on structures that were insufficiently recognised by the prior model and loaded their model predictions into QuPath to provide accurate starting points for annotation. This procedure strongly reduced annotation efforts as it converted the manual annotation into an annotation correction process. However, it was only implemented for training data, meaning that all data used to assess performance and validate the models was generated completely by experts. This cycle was repeated twice after two and three quarters of data annotations. To ensure generalisability to external cohorts and applicability in a broad spectrum of diseases, specimens from our internal cohorts that reflect the diversity of kidney histopathology for training and testing were selected (e.g., 21 cases of IgA nephropathy (IgAN), 11 pauci-immune glomerulonephritides (GN), a full list of diagnoses included in training/testing/external validation is provided in Supp. Table 17.

### Tissue and structure segmentation CNNs

For the segmentation of kidney tissue, we used a nnU-Net, representing the state-of-the-art for biomedical image segmentation^43^. For the segmentation of histological structures, we have built on our previous study on kidney structure segmentation in experimental nephropathology by employing the same U-Net-like architecture and training routine as they were specifically developed and comprehensively validated for this particular task^19^. Both CNNs were developed using the internal AC_B and AC_N cohorts while the external held-out data from the KPMP and HuBMAP cohorts were used for external validation only.

### Tissue segmentation

A nnU-Net was used for the kidney tissue segmentation task. The nnU-Net has gained popularity due to its superiority in several international biomedical segmentation competitions without the need for application-specific manual intervention^43^. For training, regions of size 2500 × 2500 µm^2^ were extracted in a grid-like manner from the annotated internal cohorts AC_B & AC_N and downsampled into patches of size 512 × 512 pixels. Regions containing at least 90% white background were ignored. A batch size of 12 was used. Soft Dice loss as well as binary cross-entropy were used as equally weighted loss functions. The initial learning rate of 0.01 was polynomially reduced using a power of 0.9 during a total amount of 1000 epochs. Colour and spatial transformations including Gaussian noise, Gaussian blur, gamma contrast, rotations and scaling were used for data augmentation. To further increase the robustness of the model, a five-fold cross-validation on the training data was performed to train an ensemble of five different convolutional neural networks (CNNs). We then selected the model providing the lowest internal validation loss as the final one in each respective fold. Given an input image, the final prediction was then obtained by averaging the predictions of all five models, followed by filling holes and removing very small predictions. Overall, 12,034 patches from 1056 WSIs were extracted and used for the development, testing and validation of the model. Training was conducted only on the internal AC_B and AC_N cohorts using a random 80% data split on patient level, respectively. The remaining 20% of data from both internal cohorts were used for testing (Supp. Table 16).

### Structure segmentation

A U-Net-like model^44^ was trained on random minibatches of size six using RAdam^45^ as an optimiser for the kidney structure segmentation task. The initial learning rate of 0.001 was divided by three in case the internal validation loss did not drop for 15 epochs straight. Training terminated once the learning rate fell below 4E-6. Then, the model providing the lowest internal validation loss was chosen as the final configuration. Soft Dice-loss as well as the weighted categorical cross-entropy (WCE) were used as equally weighted loss functions as well. In addition, affine, piecewise affine, elastic, 90-degree rotation, flipping, hue and saturation shifting, and gamma contrast transformations were used for data augmentation. This simulates variability in tissue staining and morphology for improved generalisation. Stain normalisation approaches have not shown improvement in performance^46^. This result confirmed the CNN pipeline to effectively tackle colour variability across the external cohorts. Further, CNN predictions were post-processed by first applying test-time augmentation to improve the model’s robustness, second, removing border class predictions, third, filling prediction holes, fourth, removing too small instance predictions, and fifth, dilating tubular predictions. A border class comprising border pixels of all structures to enable the separation of touching instances from the same class, and thus instance-level analysis was implemented. Border pixels were computed for tubules by dilation using a ball-shape structuring element of radius three pixels. Full borders of arteries and glomeruli were not included into the border class as this would have prevented continuous class transitions, e.g., between tubules and glomeruli at their urinary pole. Instead, arteries and glomeruli were dilated using a radius of seven pixels and only their class-specific overlap was included into the border class. Consequently, the border class mostly represented the tubular basement membranes. Using the WCE, a ten times greater weight was assigned to the border class for improved instance separation. Annotated data from the internal cohorts (AC_B: 68 WSIs, 3,162 patches; AC_N: 17 WSIs, 431 patches) was split into training (AC_B: 54 WSIs, 2,621 patches; AC_N: 10 WSIs, 200 patches), internal validation (AC_B: 2 WSIs, 78 patches; AC_N: 2 WSIs, 30 patches) and test set (AC_B: 12 WSIs, 463 patches; AC_N: 5 WSIs, 201 patches) on case level. External validation was performed on the held-out data from the KPMP and HuBMAP cohorts on 240 and 198 patches from five WSIs, respectively (Supp. Table 16). Here, external validation refers to CNN testing on external cohorts not seen during training for assessment of generalisation, while internal validation measures CNN performance on a dedicated training data split during training to improve its generalisation.

### Performance evaluation

Regular Dice-similarity-coefficients (DSCs) were used to measure performance of the tissue segmentation CNN on WSI-level. Performance of the structure segmentation CNN was assessed by instance Dice-similarity-coefficients (iDSCs) to measure accuracy specifically on instance-level. For each prediction instance, a Dice score measuring the spatial overlap with its maximally overlapping ground-truth instance was computed, which was repeated vice versa for each ground truth instance. Thus, the iDSC quantifies the mean predicted area coverage per instance, and ranges like the DSC from 0 (no single ground-truth overlap) to 1 (perfect predictions). F1-Scores and positive predictive values (PPVs) were computed by counting the true positives (correctly segmented instances defined as an iDSC above the threshold of 0.5), false positives and false negatives.

### Feature extraction

FLASH enabled us to extract 7,382,198 instances of segmented histological structures (6,742,314 tubules, 89,160 glomeruli and 550,724 arteries) from the five cohorts. Each segmented instance represents a geometrical object, enabling the quantitative assessment of 35 hand-crafted morphometric histological. We focused only on easily explainable features to facilitate interpretability. Also, due to very large divergence in staining in the multi-centre cohorts, the extraction of colour or texture-based features was not feasible. We computed the area [µm²] and diameter [µm] of the cross-section of tubules, glomeruli, glomerular tufts, arteries and their corresponding lumina. To encompass simultaneous changes in glomerular and glomerular tuft area the Tuft-Area-Fraction was calculated by dividing glomerular tuft area through glomerular area per instance as well as the Bowman’s area by subtracting the glomerular tuft area from the glomerular area. The area and diameter of whole arteries, the lumen and the wall consisting of intima and media were calculated (adventitia was not segmented and excluded). Small arterioles (full diameter <50 µm) were excluded due to lower segmentation accuracy to enable accurate morphometry analysis. Distances [µm] between glomeruli and their respective nearest glomerular neighbour, as well as between tubules and their respective nearest structure were also calculated. The circularity, eccentricity, elongation and solidity which were calculated based on common shape extraction techniques^47^, each taking values between 0 and 1 were calculated for full glomeruli and glomerular tufts. Instance counts and area percentages were extracted on WSI-level. Features were calculated in all five cohorts on instance-level but were summarised on patient-level as well. All instances inherited the diagnostic or clinical label of their parent case, facilitating the comparison of diseased cases to normal cases as well as the analysis of discrepancies between different kidney diseases.

Additional information regarding the computation of morphometrical features is given in Supp. Table 3.

### Cox proportional hazards models

Time from kidney biopsy to the composite endpoint up to 15 years (end-stage kidney disease (ESKD) and/or halving of initial eGFR assessed at time of biopsy) was analysed in the VALIGA cohort by implementing Cox proportional hazards models. 4 cases had to be excluded due to missing data (initial eGFR). eGFR and age were grouped by steps of 10. The proportional hazard assumption was assessed graphically using Schoenfeld residuals. Five features (tubular distance, tubular diameter, tuft area, tuft circularity and tuft eccentricity) which were descriptive of disease morphology or kidney function based on eGFR in our internal biopsy cohort were selected as digital biomarkers. Optimal cut-offs for all five features were determined by computing the maximally selected log-rank statistic^48^. No model based on the continuous distribution of features was computed because of the large variation in effect sizes due to different scaling (e.g., 0–1 or 0-60,000 um²). Normalisation of feature distributions was not performed due to the lacking explainability of the actual changes in histopathology. The five features were then used to fit initial unadjusted models. Next, each model was adjusted for age, sex, initial eGFR and MEST-C score (each component of the score respectively). Additionally, a Digital Biomarkers model was constructed, using all five features as predictors which was adjusted for age, sex and eGFR at the time of biopsy. In addition, a MEST-C model with the M, E, S, T and C components of the score as covariates was constructed and adjusted for age, sex and initial eGFR. A third, hybrid model combined both previously described cox proportional hazards models. Models were compared to each other based on C-Statistic, Akaike information criterion (AIC) and Bayesian information criterion (BIC).

### Single structure trajectory analysis

We used Seurat (4.1.0 version)^49^ to perform a single structure trajectory analysis. We considered structures as samples and structure features as columns. This analysis was done independently for tubules and glomeruli due to distinct features. Next, we ran NormalizeData with the parameter normalisation.method = ‘RC’ (Relative counts) to normalise each structure. We used the Corral package (version 1.4.0)^50^ to perform dimension reduction using Pearson Residuals based correspondence analysis. Next, we produced a diffusion map using the destiny package (3.8.1)^51^ with default parameters. We performed Louvain clustering with the first two components of diffusion embeddings by calling FindNeighbors and FindClusters from the Seurat package. Finally, we found trajectories using ArchR (version 1.0.1)^52^. Specifically, we first defined a backbone by selecting a list of clusters from healthy to disease and using their function addTrajectory to detect a pseudo-time scale from 0 to 100. For line plots, we distributed all patients to 20 buckets from 0 to 100 and calculated the fractions in each bucket of each condition. We next fitted smoothed lines using method loess (locally estimated scatterplot smoothing). Additionally, we explored concepts of the Wasserstein distance to obtain a patient specific pseudotime. In short, we encoded clusterings of patient’s glomeruli and tubules as probability distributions, which are compared with the Wasserstein distance (similar as in ref. ^53^). We combined the distance matrices for computing a patient specific trajectory similar to instance level data.

### Statistics and reproducibility

All statistical calculations were performed within the computing environment R (v4.0.3). We performed two-sample Anderson-Darling tests^54^ for comparison between different feature distributions. We further calculated 5000-times bootstrapped 95%-confidence intervals of difference in feature medians for group comparisons. Comparing groups with smaller sample sizes, e.g., specimen-level comparison of histopathology, we performed a Kruskal-Wallis test and two-sided pairwise Wilcoxon rank-sum tests. For multiple comparisons, e.g., across diseases, we corrected for multiple testing by Bonferroni-type adjustment of p-values in each group individually. Probabilities of progression-free survival for VALIGA biopsies were assessed by calculating Cox proportional hazards models with hazard ratios (HR) and 95% confidence intervals (see above). Categorical variables were interpreted as absolute (n) and relative (%) frequencies while descriptive continuous features were described as mean/median + IQR. Values of *p*  <  0.05 were considered significant.

CNN-based segmentation and feature extraction were reproducible, and multiple different runs of the single structure trajectory analysis pipeline produced similar embedding, clustering and trajectory results.

### Data analysis with R

Quantitative feature analyses for each WSI were imported as dataframes into the R Environment and data transformation was performed using the following publicly available packages: tidyverse v1.3.1 (cran.r-project.org/web/packages/tidyverse), dplyr v1.0.8 (cran.r-project.org/web/packages/dplyr), gdata v2.18.0 (cran.r-project.org/web/packages/gdata), and broom v0.7.12 (cran.r-project.org/web/packages/broom). Statistics were calculated using the kSamples v1.2-9 (cran.r-project.org/web/packages/kSamples), PMCMRplus v1.9.3 (cran.rstudio.com/web/packages/PMCMRplus), simpleboot v1.1-7 (https://cran.r-project.org/web/packages/simpleboot) and boot v1.3-28 (https://cran.r-project.org/web/packages/boot) packages. Survival analysis was performed with the survival v3.3-0 (cran.rstudio.com/web/packages/survival), survminer v0.4.9 (cran.r-project.org/web/packages/survminer) and maxstat v0.7-25 (cran.rstudio.com/web/packages/maxstat) packages.

Plots were created by using the packages: ggplot2 v3.3.3 (cran.r-project.org/web/packages/ggplot2) and cowplot v1.1.1 (cran.r-project.org/web/packages/cowplot).

### Reporting summary

Further information on research design is available in the Nature Portfolio Reporting Summary linked to this article.

## Supplementary information

Supplementary Information

Suppl. Tables

Reporting Summary

## Data Availability

The pathomics data, associated clinical data and many segmentation images (>2000 paired image patches (PAS plus segmentation)) generated in this study have been deposited in our github repository: https://git-ce.rwth-aachen.de/labooratory-ai/flash. The raw whole slide image data are available under restricted access for privacy protection reasons, access can be obtained by directly contacting Peter Boor, Institute of Pathology, RWTH Aachen University Clinic, Aachen, Germany, pboor@ukaachen.de (for the AC_B and AC_N datasets) or Rosanna Coppo, Fondazione Ricerca Molinette, Torino, Italy, rosanna.coppo@unito.it (for the VALIGA dataset). In general, the requests will be evaluated within 4 weeks based on institutional and trial policies. Data can only be shared for non-commercial research purposes and requires a data transfer agreement. The aggregated data and raw data used to create figure panels generated in this study are provided in the Supplementary Information/Source Data files. The public external image and clinical data used in this study are available in the KPMP (atlas.kpmp.org/repository) and HubMAP (portal.hubmapconsortium.org) databases. Source data are provided with this paper.

## Source data

Source Data

## Code availability

The source code for FLASH and instructions on how to use it are are freely available at: git-ce.rwth-aachen.de/labooratory-ai/flash.
