# Supplementary material for: Next-Generation Morphometry for pathomics-data mining in histopathology
Source: Nat Commun. 2023 Jan 28;14:470. doi: 10.1038/s41467-023-36173-0 (PMC9884209; doi:10.1038/s41467-023-36173-0)
Supplement: Supplementary file 1 — Supplementary Information [file 41467_2023_36173_MOESM1_ESM.pdf]

Supplementary Material to:
Next-Generation Morphometry for pathomics-data mining in histopathology

Running title: Automated segmentation and quantification of human kidney histopathology

David L. Hölscher[1,#], Nassim Bouteldja[1,#], Mehdi Joodaki[2], Maria L. Russo[3], Yu-Chia Lan[1], Alireza Vafaei Sadr[1], Mingbo Cheng[2], Vladimir Tesar[4], Saskia v. Stillfried[1], Barbara M. Klinkhammer[1], Jonathan Barratt[5,6], Jürgen Floege[7], Ian SD Roberts[8], Rosanna Coppo[3,9], Ivan G. Costa[2], Roman D. Bülow[1,§], Peter Boor[1,7,§,*]

1 Institute of Pathology, RWTH Aachen University Clinic, Aachen, Germany
2 Institute for Computational Genomics, RWTH Aachen University Clinic, Aachen, Germany
3 Fondazione Ricerca Molinette, Torino, Italy
4 Department of Nephrology, 1st Faculty of Medicine and General University Hospital, Charles University, Prague, Czech Republic
5 John Walls Renal Unit, University Hospital of Leicester National Health Service Trust, Leicester, United Kingdom
6 Department of Cardiovascular Sciences, University of Leicester, Leicester, United Kingdom
7 Department of Nephrology and Immunology, RWTH Aachen University Clinic, Aachen, Germany
8 Department of Cellular Pathology, Oxford University Hospitals National Health Services Foundation Trust, Oxford, United Kingdom
9 Regina Margherita Children's University Hospital, Torino, Italy

# These authors contributed equally
§ These authors jointly supervised this work

* Address correspondence to:
Peter Boor, M.D., Ph.D.
Institute of Pathology
RWTH Aachen University Hospital
Pauwelsstrasse 30
52074 Aachen, Germany
Phone: +49 241 80 85227
Fax: +49 241 80 82446
E-mail: pboor@ukaachen.de

Table of Contents

Group Information

Supp. Table 1: Clinical and general patient data for the five datasets

Supp. Table 2: Annotation criteria consensus

Supp. Table 3: Description of quantitative morphometric features

Supp. Table 4: Quantitative analysis of glomerular and tubular morphometry in native kidney diseases

Supp. Table 5: Quantitative analysis of glomerular morphometry based on proteinuria

Supp. Table 6: Quantitative analysis of glomerular and tubular morphometry based on kidney function

Supp. Table 7: Quantitative analysis of tubular morphometry based on scored histopathology of interstitial fibrosis and tubular atrophy

Supp. Table 8: Quantitative analysis of vascular morphometry based on scored arteriosclerosis and reported hypertension status

Supp. Table 9: Cumulative events in univariate Cox proportional hazards models

Supp. Table 10: Uni- & multivariate Cox proportional hazards models

Supp. Table 11: Digital biomarkers & MEST-C Cox regression models

Supp. Table 12: Clinical and histopathological characteristics of multivariate Cox proportional hazards models

Supp. Table 13: Data availability for internal and external cohorts

Supp. Table 14: Diagnoses/diseases present in the AC_B cohort

Supp. Table 15: Annotated instances of histological structures

Supp. Table 16: Overview of training, testing and validation data

Supp. Table 17: Diagnoses/diseases present in training, testing and validation data

Supp. Figure 1: Kidney tissue segmentation visualisations

Supp. Figure 2: Stain variation between the five cohorts

Supp. Figure 3: Structure segmentation visualisations from internal and external cohorts

Supp. Figure 4: Visualisation of incorrect structure segmentations

Supp. Figure 5: Disease comparison for various features

Supp. Figure 6: Vascular morphometry based on scored arteriosclerosis

Supp. Figure 7: Glomerular phenotypes along pseudotime trajectory

Supp. Figure 8: Pseudotime analysis of NGM-derived glomerular and tubular features aggregated on patient-level

Supp. Figure 9: Visualisation of feature outliers

Supplementary References

Group information

The International IgA Nephropathy Network members are as follows:
VALIGA investigators: M.L. Russo (MA, PhD, Fondazione Ricerca Molinette, Torino, Italy); S. Troyanov (MD, Division of Nephrology, Department of Medicine, Hopital du Sacre-Coeur de Montreal, Montreal, Quebec, Canada); H.T. Cook (MD, Centre for Complement and Inflammation Research, Department of Medicine, Imperial College, London, England); I. Roberts (MD, Department of Cellular Pathology, Oxford University Hospitals NHS Foundation Trust, John Radcliffe Hospital, Oxford, United Kingdom); V. Tesar, (MD, Department of Nephrology, 1st Faculty of Medicine and General University Hospital, Charles University, Prague, Czech Republic); D. Maixnerova (MD, Department of Nephrology, 1st Faculty of Medicine and General University Hospital, Charles University, Prague, Czech Republic); S. Lundberg (MD, Nephrology Unit, Department of Clinical Sciences, Karolinska Institute, Stockholm, Sweden); L. Gesualdo (MD, Department of Nephrology, Emergency and Organ Transplantation, University of Bari “Aldo Moro,” Foggia-Bari, Italy); F. Emma (MD, Division of Nephrology, Department of Pediatric Subspecialties, Bambino Gesù Children’s Hospital IRCCS, Rome, Italy); F. Diomedi (MD, Division of Nephrology, Department of Pediatric Subspecialties, Bambino Gesù Children’s Hospital IRCCS, Rome, Italy); G. Beltrame (MD, Nephrology and Dialysis Unit, San Giovanni Bosco Hospital, and University of Turin,Turin, Italy); C. Rollino (MD, Nephrology and Dialysis Unit, San Giovanni Bosco Hospital, and University of Turin,Turin, Italy); A. Amore (MD, Nephrology Unit, Regina Margherita Children’s Hospital,Turin, Italy); R. Camilla (MD Nephrology Unit, Regina Margherita Children’s Hospital, Turin, Italy); L. Peruzzi (MD, Nephrology Unit, Regina Margherita Children’s Hospital, Turin, Italy); M. Praga (MD, Nephrology Unit, Hospital 12 de Octubre,Madrid, Spain); S. Feriozzi (MD, Nephrology Unit, Belcolle Hospital, Viterbo, Italy), R. Polci, (MD, Nephrology Unit, Belcolle Hospital,Viterbo, Italy); G. Segoloni, (MD, Division of Nephrology Dialysis and Transplantation, Department of Medical Sciences, Città della Salute e della Scienza Hospital and University of Turin, Turin, Italy); L.Colla (MD, Division of Nephrology Dialysis and Transplantation, Department of Medical Sciences, Città della Salute e della Scienza Hospital and University of Turin,Turin, Italy); A. Pani (MD, Nephrology Unit, G. Brotzu Hospital, Cagliari, Italy); D. Piras (MD, Nephrology Unit, G. Brotzu Hospital, Cagliari, Italy), A. Angioi (MD, Nephrology Unit, G. Brotzu Hospital, Cagliari, Italy); G. Cancarini, (MD, Nephrology Unit, Spedali Civili University Hospital, Brescia, Italy); S. Ravera (MD, Nephrology Unit, Spedali Civili University Hospital, Brescia, Italy); M. Durlik (MD, Department of Transplantation Medicine, Nephrology, and Internal Medicine, Medical University ofWarsaw,Warsaw, Poland); E. Moggia (Nephrology Unit, Santa Croce Hospital, Cuneo, Italy); J. Ballarin (MD, Department of Nephrology, Fundacion Puigvert, Barcelona, Spain); S. Di Giulio (MD, Nephrology Unit, San Camillo Forlanini Hospital, Rome, Italy); F. Pugliese (MD, Department of Nephrology, Policlinico Umberto I University Hospital, Rome, Italy); I. Serriello (MD, Department of Nephrology, Policlinico Umberto I University Hospital, Rome, Italy); Y. Caliskan (MD, Division of Nephrology, Department of Internal Medicine, Istanbul Faculty of Medicine, Istanbul University, Istanbul, Turkey); M. Sever (MD, Division of Nephrology, Department of Internal Medicine, Istanbul Faculty of Medicine, Istanbul University, Istanbul, Turkey); I. Kilicaslan (MD, Department of Pathology, Istanbul Faculty of Medicine, Istanbul University, Istanbul, Turkey); F. Locatelli (MD, Department of Nephrology and Dialysis, Alessandro Manzoni Hospital, ASST

Lecco, Italy); L. Del Vecchio (MD, Department of Nephrology and Dialysis, Alessandro Manzoni Hospital, ASST Lecco, Italy); J.F.M.Wetzels (MD, Departments of Nephrology, Radboud University Medical Center, Nijmegen, the Netherlands); H. Peters (MD, Departments of Nephrology, Radboud University Medical Center, Nijmegen, the Netherlands); U. Berg (MD, Division of Pediatrics, Department of Clinical Science, Intervention and Technology, Huddinge, Sweden); F. Carvalho (MD, Nephrology Unit, Hospital de Curry Cabral, Lisbon, Portugal); A.C. da Costa Ferreira (MD, Nephrology Unit, Hospital de Curry Cabral, Lisbon, Portugal); M. Maggio (MD, Nephrology Unit, Hospital Maggiore di Lodi, Lodi, Italy); A. Wiecek (MD, Department Nephrology, Endocrinology and Metabolic Diseases, Silesian University of Medicine, Katowice, Poland); M. Ots-Rosenberg(MD, Nephrology Unit, Tartu University Clinics, Tartu,Estonia); R. Magistroni (MD, Department of Nephrology, Policlinic of Modena and Reggio Emilia; Modena, Italy); R. Topaloglu (MD, Department of Pediatric Nephrology and Rheumatology, Hacettepe University, Ankara, Turkey); Y. Bilginer (MD, Department of Pediatric Nephrology and Rheumatology, Hacettepe University, Ankara, Turkey); M. D'Amico (MD, Nephrology Unit, S. Anna Hospital, Como, Italy); M. Stangou (MD, Department of Nephrology, Hippokration General Hospital, Aristotle University of Thessaloniki, Thessaloniki, Greece); F. Giacchino (MD, Nephrology Unit, Ivrea Hospital, Ivrea, Italy); D. Goumenos (MD Department of Nephrology, University Hospital of Patras, Patras, Greece); P. Kalliakmani (MD Department of Nephrology, University Hospital of Patras, Patras, Greece); M. Papasotiriou (MD Department of Nephrology, University Hospital of Patras, Patras, Greece); K. Galesic (MD, Department of Nephrology, University Hospital Dubrava, Zagreb, Croatia); C. Geddes (MD, Renal Unit,Western Infirmary Glasgow, Glasgow, United Kingdom); K. Siamopoulos (MD, Nephrology Unit, Medical School University of Ioanina, Ioannina, Greece); O. Balafa (MD, Nephrology Unit,Medical School University of Ioanina, Ioannina, Greece); M. Galliani (MD, Nephrology Unit, S.Pertini Hospital, Rome, Italy); P. Stratta (MD, Department of Nephrology, Maggiore della Carità Hospital, Piemonte Orientale University, Novara, Italy); M. Quaglia (MD, Department of Nephrology, Maggiore della Carità Hospital, Piemonte Orientale University, Novara, Italy); R. Bergia (MD, Nephrology Unit, Degli Infermi Hospital, Biella,Italy); R. Cravero (MD, Nephrology Unit, Degli Infermi Hospital, Biella, Italy); M. Salvadori, (MD, Department of Nephrology, Careggi Hospital, Florence, Italy); L. Cirami (MD, Department of Nephrology, Careggi Hospital, Florence, Italy); B. Fellstrom (MD, Renal Department, University of Uppsala, Uppsala, Sweden); H. Kloster Smerud (MD, Renal Department, University of Uppsala, Uppsala, Sweden); F. Ferrario (MD, Nephropathology Unit, San Gerardo Hospital, Monza, Italy); T. Stellato (MD, Nephropathology Unit, San Gerardo Hospital, Monza, Italy); J. Egido (MD, Department of Nephrology, Fundacion Jimenez Diaz, Madrid, Spain); C. Martin (MD, Department of Nephrology, Fundacion Jimenez Diaz, Madrid, Spain); J. Floege (MD, Nephrology and Immunology, Medizinische Klinik II, University of Aachen, Aachen, Germany); F. Eitner (MD, Nephrology and Immunology, Medizinische Klinik II, University of Aachen, Aachen, Germany); A. Lupo (MD, Department of Nephrology, University of Verona, Verona, Italy); P. Bernich (MD, Department of Nephrology, University of Verona, Verona, Italy); P. Menè (Department of Nephrology,S. Andrea Hospital, Rome, Italy); M. Morosetti (Nephrology Unit, Grassi Hospital, Ostia, Italy); C. van Kooten, (MD, Department of Nephrology, Leiden University Medical Centre, Leiden, The Netherlands); T. Rabelink (MD, Department of Nephrology, Leiden University Medical Centre, Leiden, The Netherlands); M.E.J. Reinders (MD, Department of Nephrology, Leiden University Medical Centre, Leiden, The Netherlands); J.M. Boria Grinyo (Department of Nephrology, Hospital Bellvitge, Barcelona, Spain); S. Cusinato (MD, Nephrology Unit, Borgomanero Hospital, Borgomanero, Italy); L. Benozzi (MD, Nephrology Unit, Borgomanero Hospital, Borgomanero, Italy); S.

Savoldi, (MD, Nephrology Unit, Civile Hospital, Ciriè, Italy); C. Licata (MD, Nephrology Unit, Civile Hospital, Ciriè, Italy); M. Mizerska-Wasiak (MD, Department of Pediatrics, Medical University ofWarsaw,Warsaw, Poland); G. Martina (MD, Nephrology Unit, Chivasso Hospital, Chivasso, Italy); A. Messuerotti (MD, Nephrology Unit, Chivasso Hospital, Chivasso, Italy); A. Dal Canton (MD, Nephrology Unit, S. Matteo Hospital, Pavia, Italy); C. Esposito (MD, Nephrology Unit, Maugeri Foundation, Pavia, Italy); C. Migotto (MD, Nephrology Unit, Maugeri Foundation, Pavia, Italy); G. Triolo MD, Nephrology Unit CTO, Turin, Italy); F.Mariano (MD, Nephrology Unit CTO, Turin, Italy); C. Pozzi (MD, Nephrology Unit, Bassini Hospital, Cinisello Balsamo, Italy); R. Boero (MD, Nephrology Unit, Martini Hospital, Turin, Italy);

VALIGA pathology investigators: S. Bellur (MD, Department of Cellular Pathology, Oxford University Hospitals NHS Foundation Trust, John Radcliffe Hospital, Oxford, United Kingdom); G.Mazzucco (MD, Pathology Department, University of Turin, Turin, Italy); C. Giannakakis (MD, Pathology Department, La Sapienza University, Rome, Italy); E. Honsova (MD, Department of Clinical and Transplant Pathology, Institute for Clinical and Experimental Medicine, Prague, Czech Republic); B. Sundelin (MD Department of Pathology and Cytology, Karolinska University Hospital, Karolinska Institute, Stockholm, Sweden); A.M. Di Palma (Nephrology Unit, Aldo Moro University, Foggia-Bari, Italy); F. Ferrario (MD, Nephropathology Unit, San Gerardo Hospital, Monza, Italy); E. Gutiérrez (MD, Renal, Vascular and Diabetes Research Laboratory, Fundación Instituto de Investigaciones Sanitarias-Fundación Jiménez Díaz, Universidad Autónoma de Madrid, Madrid, Spain); A.M. Asunis (MD, Department of Pathology, Brotzu Hospital, Cagliari, Italy); J. Barratt (MD, The JohnWalls Renal Unit, Leicester General Hospital, Leicester, United Kingdom); R. Tardanico (MD, Department of Pathology, Spedali Civili Hospital, University of Brescia, Brescia, Italy); A. Perkowska-Ptasinska (MD, Department of Transplantation Medicine, Nephrology and Internal Medicine, Medical University of Warsaw, Warsaw, Poland); J. Arce Terroba (MD, Pathology Department, Fundació Puigvert, Barcelona, Spain); M. Fortunato (MD, Pathology Department, S. Croce Hospital, Cuneo, Italy); A. Pantzaki (MD, Department of Pathology, Hippokration Hospital, Thessaloniki, Greece); Y. Ozluk (MD, Department of Pathology, Istanbul University, Istanbul Faculty of Medicine, Istanbul, Turkey); E. Steenbergen (MD, Radboud University Medical Center, Department of Pathology, Nijmegen, The Netherlands); M. Soderberg (MD, Department of Pathology, Drug Safety and Metabolism, Huddinge, Sweden); Z. Riispere (MD, Department of Pathology, University of Tartu, Tartu, Estonia); L. Furci (MD, Pathology Department, University of Modena, Italy); D. Orhan (MD, Department of Pediatrics, Division of Rheumatology, Hacettepe University Faculty of Medicine, Ankara, Turkey); D. Kipgen (MD,Pathology Department, Queen Elizabeth University Hospital, Glasgow, United Kingdom); D. Casartelli (Pathology Department, Manzoni Hospital, Lecco, Italy); D. Galesic Ljubanovic (MD, Nephrology Department, University Hospital, Zagreb, Croatia; Zagreb, Croatia); H Gakiopoulou (MD, Department of Pathology, National and Kapodistrian University of Athens, Athens, Greece); E. Bertoni (MD, Nephrology Department, Careggi Hospital, Florence, Italy); P. Cannata Ortiz (MD, Pathology Department, IIS-Fundacion Jimenez Diaz UAM, Madrid, Spain); H. KarkoszkaMD, (Nephrology, Endocrinology and Metabolic Diseases, Medical University of Silesia, Katowice, Katowice, Poland); H.J. Groene (MD, Cellular and Molecular Pathology, German Cancer Research Center, Heidelberg, Germany); A. Stoppacciaro (MD, Surgical Pathology Units, Department of Clinical and Molecular Medicine, Ospedale Sant'Andrea, Sapienza University of Rome, Rome, Italy); I. Bajema (MD, Department of Pathology, Leiden University Medical Center, Leiden, The Netherlands); J. Bruijn (MD, Department of Pathology, Leiden University Medical Center, Leiden, The Netherlands); X. Fulladosa

Oliveras (MD, Nephrology Unit, Bellvitge University Hospital, Hospitalet de Llobregat, Barcelona, Spain); J. Maldyk (MD, Division of Pathomorphology, Children’s Clinical Hospital, Medical University ofWarsaw,Warsaw, Poland); and E. Ioachim (MD, Department of Pathology, Medical School, University of Ioannina, Ioannina, Greece); the Oxford derivation and North American validation investigators: Bavbek N (MD, Department of Pathology, Vanderbilt University, Nashville, Tennessee); Cook T (MD, Imperial College, London, England), Troyanov S (MD, Division of Nephrology, Department of Medicine, Hopital du Sacre-Coeur de Montreal, Montreal, Quebec, Canada); Alpers C (MD, Department of Pathology, University of Washington Medical Center, Seattle,Washington), Amore A (MD, Nephrology, Dialysis and Transplantation Unit, Regina Margherita Children’s Hospital, University of Turin, Turin, Italy), Barratt J (MD, The John Walls Renal Unit, Leicester General Hospital, Leicester, England); Berthoux F (MD, Department of Nephrology, Dialysis, and Renal Transplantation, Hôpital Nord, CHU de Saint-Etienne, Saint-Etienne, France); Bonsib S (MD, Department of Pathology, LSU Health Sciences Center, Shreveport, Los Angeles); Bruijn J (MD, Department of Pathology, Leiden University Medical Center, Leiden, The Netherlands); D’Agati V (MD, Department of Pathology, Columbia University College of Physicians & Surgeons, New York, New York); D’Amico G (MD, Fondazione D’Amico per la Ricerca sulle Malattie Renali, Milan, Italy); Emancipator S (MD, Department of Pathology, Case Western Reserve University, Cleveland, Ohio); Emmal F (MD, Division of Nephrology and Dialysis, Department of Nephrology and Urology, Bambino Gesù Children’s Hospital and Research Institute, Piazza S Onofrio, Rome, Italy); Ferrario F (MD, Renal Immunopathology Center, San Carlo Borromeo Hospital, Milan, Italy); Fervenza F (MD PhD, Division of Nephrology and Hypertension, Mayo Clinic, Rochester); Florquin S (MD, Department of Pathology, Academic Medical Center, University of Amsterdam, Amsterdam, The Netherlands); Fogo A (MD, Department of Pathology, Vanderbilt University, Nashville, Tennessee); Geddes C (MD, The Renal Unit, Western Infirmary, Glasgow, Scotland); Groene H (MD, Department of Cellular and Molecular Pathology, German Cancer Research Center, Heidelberg, Germany); Haas M(MD, Department of Pathology and Laboratory Medicine, Cedars-Sinai Medical Center, Los Angeles, California); Hill P (MD, St Vincent’s Hospital, Melbourne, Australia); Hogg R (MD, Scott and White Medical Center, Temple, Texas (retired)); Hsu S (MD, Division of Nephrology, Hypertension and Renal Transplantation, College of Medicine, University of Florida, Gainesville, Florida); Hunley T (MD, Department of Pathology, Vanderbilt University, Nashville, Tennessee); Hladunewich (MD, Division of Nephrology, Sunnybrook Health Science Center, University of Toronto, Ontario, Canada M); Jennette C (MD, Department of Pathology and Laboratory Medicine, University of North Carolina, Chapel Hill, North Carolina); Joh K (MD, Division of Immunopathology, Clinical Research Center Chiba, East National Hospital, Chiba, Japan); Julian B (MD, Department of Medicine, University of Alabama at Birmingham, Birmingham, Alabama); Kawamura T (MD, Division of Nephrology and Hypertension, Jikei University School of Medicine, Tokyo, Japan); Lai F (MD, The Chinese University of Hong Kong, Hong Kong); Leung C (MD, Department of Medicine, Prince of Wales Hospital, Chinese University of Hong Kong, Hong Kong); Li L (MD, Research Institute of Nephrology, Jinling Hospital, Nanjing University School of Medicine, Nanjing, China); Li P (MD, Department of Medicine, Prince of Wales Hospital, Chinese University of Hong Kong, Hong Kong); Liu Z (MD, Research Institute of Nephrology, Jinling Hospital, Nanjing University School of Medicine, Nanjing, China); Massat A (MD, Division of Nephrology and Hypertension, Mayo Clinic, Rochester, Minnesota); Mackinnon B (MD, The Renal Unit,Western Infirmary, Glasgow, Scotland); Mezzano S (MD, Departamento de Nefrología, Escuela de Medicina, Universidad

Austral, Valdivia, Chile); Schena F (MD, Renal, Dialysis and Transplant Unit, Policlinico, Bari, Italy); Tomino Y (MD, Division of Nephrology, Department of Internal Medicine, Juntendo University School of Medicine, Tokyo, Japan); Walker P (MD, Nephropathology Associates, Little Rock, Arkansas);Wang H (MD, Renal Division of Peking University First Hospital, Peking University Institute of Nephrology, Beijing, China (deceased)); Weening J (MD, Erasmus Medical Center, Rotterdam, The Netherlands); and Yoshikawa N (MD, Department of Pediatrics, Wakayama Medical University, Wakayama City, Japan); the International investigators: Cai-Hong Zeng (MD, Nanjing University School of Medicine, Nanjing, China); Sufang Shi (MD, Peking University Institute of Nephrology, Beijing, China); C.Nogi (MD, Juntendo University, Faculty of Medicine, Tokyo, Japan); H.Suzuki (MD, Juntendo University, Faculty of Medicine, Tokyo, Japan); K. Koike (MD, Division of Nephrology and Hypertension, Department of Internal Medicine, Jikei University School of Medicine, Tokyo, Japan); K. Hirano (MD, Division of Nephrology and Hypertension, Department of Internal Medicine, Jikei University School of Medicine, Tokyo, Japan); T. Kawamura (MD, Division of Nephrology and Hypertension, Department of Internal Medicine, Jikei University School of Medicine, Tokyo, Japan); T. Yokoo (MD, Division of Nephrology and Hypertension, Department of Internal Medicine, Jikei UniversitySchool of Medicine, Tokyo, Japan); M. Hanai (MD, Division of Nephrology, Department of Medicine, Kurume University School of Medicine, Fukuoka, Japan); K. Fukami (MD, Division of Nephrology, Department of Medicine, Kurume University School of Medicine, Fukuoka,, Japan); K. Takahashi (MD, Department of Nephrology, Fujita Health University School of Medicne, Aichi, Japan); Y. Yuzawa (MD, Department of Nephrology, Fujita Health University School of Medicine, Aichi, Japan); M. Niwa (MD, Department of Nephrology, Nagoya University Graduate School of Medicine, Aichi, Japan); Y. Yasuda (MD, Department of Nephrology, Nagoya University Graduate School of Medicine, Aichi, Japan); S. Maruyama (MD, Department of Nephrology, Nagoya University Graduate School of Medicine, Aichi, Japan); D. Ichikawa (MD, Division of Nephrology and Hypertension, Department of Internal Medicine, St Marianna University School of Medicine, Kanagawa, Japan); T. Suzuki (MD, Division of Nephrology and Hypertension, Department of Internal Medicine, St Marianna University School of Medicine, Kanagawa, Japan); S. Shirai (MD, Division of Nephrology and Hypertension, Department of Internal Medicine, St Marianna University School of Medicine, Kanagawa, Japan); A. Fukuda (MD, First Department of Internal Medicine, Faculty of Medicine, University of Miyazaki, Miyazaki, Japan); S. Fujimoto (MD, Department of Hemovascular Medicine and Artificial Organs, Faculty of Medicine, University of Miyazaki, Miyazaki, Japan); H. Trimarchi (MD, Division of Nephrology, Hospital Britanico, Buenos Aires, Argentina).

Supplementary Table 1. Clinical, general patient data and details on the number of instances as well as measurements for included cases across the five datasets. AC_B and AC_N are internal cohorts while KPMP, HuBMAP and VALIGA are external cohorts. Instances represent one singular structure from a whole-slide image and measurements refer to the amount of calculated features per all instances (17 features for glomeruli, 5 features for tubules and 11 features for arteries). In the internal cohorts presence of hypertension regardless of its aetiology was evaluated by ICD-10 coded diseases. Age, estimated glomerular filtration rate and proteinuria are reported as mean ± one standard deviation.
F: Female; M: Male; eGFR, estimated glomerular filtration rate; G1, eGFR ≥90ml/min/1.73m²; G2, eGFR 60-89ml/min/1.73m²; G3, eGFR 30-59ml/min/1.73m²; G4, eGFR 15-29ml/min/1.73m²; G5, eGFR <15ml/min/1.73m².

Cohort	Cases [n]	Instances	Measurements	Age	Sex	eGFR [mg/dl/1.73m²]	Proteinuria [mg/d]	Hypertension [y/n]
AC_B	320	Glomeruli: 31,246<br>Tubules: 2,386,929<br>Arteries: 169,397	Glomeruli: 437,444<br>Tubules: 4,773,858<br>Arteries: 338,794	51.78<br>(±16.06)	F: 129 (40.3%)<br>M: 191 (59.7%)	39.75<br>(±31.49)	2,719.20<br>(±4,458.70)	y = 71.4%<br>(n=202)
AC_N	30	Glomeruli: 16,847<br>Tubules: 1,376,334<br>Arteries: 178,847	Glomeruli: 235,858<br>Tubules: 2,752,668<br>Arteries: 357,694	57.97<br>(±18.22)	F: 16 (53.3%)<br>M: 14 (46.7%)	NA	NA	y = 80.0%<br>(n=24)
KPMP	36	Glomeruli: 3,321<br>Tubules: 211,287<br>Arteries: 18,538	Glomeruli: 46,494<br>Tubules: 422,574<br>Arteries: 37,076	20-29: 1 (2.8%)<br>30-39: 4 (11.1%)<br>40-49: 3 (8.3%)<br>50-59: 5 (13.9%)<br>60-69: 18 (50.0%)<br>70-79: 5 (13.9%)	F: 21 (58.3%)<br>M: 15 (41.7%)	G1: 6 (17.7%)<br>G2: 8 (23.5%)<br>G3: 17 (50.0%)<br>G4: 3 (8.8%)	<150: 4 (21.1%)<br>150-499: 5 (26.3%)<br>500-999: 3 (15.8%)<br>≥1,000: 7 (36.8%)	y = 79.4%<br>(n=27)
HuBMAP	9	Glomeruli: 5,898<br>Tubules: 365,579<br>Arteries: 23,719	Glomeruli: 82,572<br>Tubules: 731,158<br>Arteries: 47,438	58.56<br>(±13.10)	F: 4 (44.4%)<br>M: 5 (55.6%)	NA	NA	y = 55.6%<br>(n=5)
VALIGA	648	Glomeruli: 31,848<br>Tubules: 2,402,185<br>Arteries: 160,223	Glomeruli: 445,872<br>Tubules: 4,804,370<br>Arteries: 320,446	36.07<br>(±15.17)	F: 182 (28.1%)<br>M: 466 (71.9%)	72.39<br>(±29.95)	1,970.97<br>(±2,039.13)	NA

Supplementary Table 2. Consensus criteria to annotate the six defined classes included in the structure segmentation.
FSGS: Focal segmental glomerulosclerosis.

Class	Annotation Criteria
Tubule	• Annotation along, but excluding the basement membrane<br>• Annotation along the inner part of split basement membrane segments
Glomerulus	• Annotation along the Bowman‘s capsule including the basement membrane<br>• Annotation along the inner part in cases with split Bowman’s capsule<br>• In cross-sections where urinary or vascular pole were visible, Annotations were continued in a straight line at the capsule border
Glomerular Tuft	• Subclass of the glomerulus class with annotation of the glomerular tuft only (including podocytes)<br>• In glomeruli with global glomerulosclerosis and no visible capillaries no tuft was annotated<br>• Extracapillary proliferates (=crescents) or FSGS tip lesions were not annotated as tuft
Non-Tissue Background	• Annotation of large white areas including veins, section background and renal pelvis<br>• For veins only the lumen was annotated, endothelium was excluded<br>• Annotations were only performed for structures with a diameter of at least 30µm
Artery	• Annotation of intima and media of arteries excluding adventitia<br>• Minimum of one visible vascular smooth muscle cell layer was required for annotation
Arterial Lumen	• Subclass of the artery class<br>• Annotation of the lumen only (including intravascular cells), endothelium was excluded

Supplementary Table 3. Description of morphometric features extracted from predicted structures by the structure segmentation convolutional neural network.
WSI: Whole-slide image.

Feature	Description
Area A	Area of the segmented structure [µm²]
Diameter $dmax$	Diameter of the largest circle fully fitting inside the structure [µm]
Distance $distmin$	Closest distance between structures of the same class [µm]
Circularity C	Measures how circular the structure is using the ratio of its area (A) multiplied by 4*π to its squared perimeter (P). The circularity of a circle equals 1. $$C = \frac{4 * \pi * A}{P^2}$$
Elongation Elo	Function of the length of the structure's minor and major axis. The elongation of a circle is 0 and gets higher the more elongated the structure is. $$Elo = 1 - \frac{minor\_axis\_length}{major\_axis\_length}$$
Eccentricity Ecc	Ratio of the distance between the structure's focal points over the major axis' length. The eccentricity equals 0 for a circle and 1 for ellipses. $Ecc = \frac{\sqrt{(major\_axis\_length^2 - minor\_axis\_length^2)}}{major\_axis\_length}$
Solidity S	Measures the density of the structure by taking the ratio of its area to the area of its convex hull H $S = \frac{A}{H}$
Area Percentage	Proportion of a class' total area from the overall tissue area [%]
Count	Number of instances of a particular class present in one WSI or specimen

Supplementary Table 4. Quantitative analysis of glomerular and tubular morphometry in common native kidney diseases from our internal biopsy cohort (AC_B). Continuous feature distributions are reported as median with interquartile range in brackets while age is reported as mean ± one standard deviation.
IgAN: IgA nephropathy; MCD: Minimal change disease; Lupus: Lupus nephritis; Membranous: Membranous glomerulonephritis; Pauci: Pauci-immune glomerulonephritis, DN: Diabetic nephropathy; HTN: Hypertensive nephropathy; F: Female.

Class	Cases (Instances)	Age	Sex	Glomerular Area [µm²]	Tuft Area [µm²]	Tuft Circularity	Tubular Diameter [µm]
Normal	17 (Glomeruli: 1,967 Tufts: 1,580 Tubules: 132,898)	49.82 (±18.55)	F: 7 (41.1%)	16,897.73 (17,307.55)	13,425.64 (12,463.40)	0.45 (0.15)	30.79 (18.34)
IgAN	44 (Glomeruli: 3,987 Tufts: 2,981 Tubules: 301,989)	42.05 (±16.6)	F: 14 (31.8%)	15,006.39 (16,601.41)	12,047.98 (12,848.73)	0.39 (0.18)	29.38 (20.41)
MCD	16 (Glomeruli: 1,784 Tufts: 1,415 Tubules: 125,706)	51.38 (±17.11)	F: 5 (31.3%)	18,235.09 (19,933.58)	15,962.92 (15,162.28)	0.41 (0.16)	31.95 (19.78)
Lupus	12 (Glomeruli: 1,500 Tufts: 1,157 Tubules: 117,257)	39.83 (±15.75)	F: 8 (66.7%)	18,149.40 (23,645.80)	16,071.97 (18,695.50)	0.38 (0.18)	29.28 (19.04)
Membranous	8 (Glomeruli: 806 Tufts: 636 Tubules: 68,849)	54.38 (±19.61)	F: 5 (62.5%)	21,157.73 (25,670.11)	18,867.77 (18,320.57)	0.41 (0.18)	31.18 (20.43)
Pauci	30 (Glomeruli: 3,498 Tufts: 2,588 Tubules: 299,736)	58.13 (±13.83)	F: 7 (23.3%)	16,900.64 (18,243.61)	11,778.88 (12,676.00)	0.36 (0.20)	29.67 (18.57)
DN	5 (Glomeruli: 723 Tufts: 330 Tubules: 48,387)	56.4 (±21.2)	F: 0 (0%)	13,416.03 (17,739.20)	11,121.69 (14,969.37)	0.34 (0.19)	25.85 (17.62)
HTN	6 (Glomeruli: 722 Tufts: 390 Tubules: 38,267)	48.67 (±9.5)	F: 1 (16.7%)	12,474.17 (21,559.04)	13,651.64 (20,009.59)	0.37 (0.19)	26.23 (20.69)

Supplementary Table 5. Quantitative analysis of glomerular morphometry based on proteinuria in our internal biopsy cohort (AC_B) and external KPMP cohort. Continuous feature distributions are reported as median with interquartile range in brackets while age is reported as mean ± one standard deviation.
MCD: Minimal change disease; Membranous: Membranous glomerulonephritis; F: Female.

Class	Cases (Instances)	Age	Sex	Tuft Area [µm²]	Tuft Circularity
AC_B					
< 3.5g/d	51 (4,839)	43.94 (±17.88)	F: 19 (37.3%)	13,026.12 (13.429.45)	0.40 (0.18)
> 3.5g/d	24 (1,606)	53.0 (±15.14)	F: 7 (29.2%)	14,290.70 (18,988.11)	0.35 (0.18)
MCD					
< 3.5g/d	4 (329)	42.5 (±20.11)	F: 1 (25.0%)	18,448.18 (13,204.64)	0.41 (0.13)
> 3.5g/d	3 (308)	62.0 (±16.46)	F: 2 (66.7%)	17,942.00 (20,155.83)	0.40 (0.14)
Membranous					
< 3.5g/d	2 (186)	33.0 (±8.49)	F: 2 (100.0%)	16,266.41 (11,756.31)	0.46 (0.14)
> 3.5g/d	2 (143)	67.5 (±20.51)	F: 2 (100.0%)	24,563.42 (21,967.46)	0.37 (0.18)
KPMP					
< 1g/d	12 (662)	30-39: 3<br>50-59: 2<br>60-69: 6<br>70-79: 1	F: 6 (50.0%)	12,537.56 (15,241.31)	0.42 (0.18)
> 1g/d	7 (379)	30-39: 1<br>40-49: 1<br>50-59: 1<br>60-69: 1<br>70-79: 3	F: 6 (85.7%)	14,882.10 (22,825.07)	0.35 (0.21)

Supplementary Table 6. Quantitative analysis of glomerular and tubular morphometry based on kidney function measured by estimated glomerular filtration rate of native biopsy cases from the AC_B cohort. Continuous feature distributions are reported as median with interquartile range in brackets while age is reported as mean ± one standard deviation.
eGFR: estimated glomerular filtration rate; F: Female.

eGFR [ml/min/1.73m²]	Cases (Instances)	Age	Sex	Tuft Circularity	Tubular Diameter [µm]	Tubular Distance [µm]
> 60	45 (Tufts: 3,818 Tubules: 355,987)	38.38 (±14.9)	F: 19 (42.2%)	0.42 (0.16)	30.47 (19.47)	3.24 (0.99)
30-60	48 (Tufts: 3,498 Tubules: 357,576)	51.69 (±15.07)	F: 18 (37.5%)	0.39 (0.17)	29.67 (20.24)	4.53 (1.45)
< 30	65 (Tufts: 5,200 Tubules: 559,540)	59.01 (±14.58)	F: 17 (26.2%)	0.36 (0.20)	28.69 (19.96)	6.04 (2.55)

Supplementary Table 7. Quantitative analysis of tubular morphometry based on scored histopathology of all cases from our internal biopsy cohort (AC_B). Structures from biopsy cases were grouped based on stratified reported scoring into four groups. Continuous feature distributions are reported as median with interquartile range in brackets while age is reported as mean ± one standard deviation.
IFTA: Interstitial fibrosis and tubular atrophy; F: Female.

IFTA	Cases (Instances)	Age	Sex	Tubular Diameter [µm]	Tubular Distance [µm]
0-10% (none/marginal)	219 (1,680,499)	51.13 (±16.49)	F: 93 (42.5%)	30.47 (19.63)	4.02 (2.10)
11-25% (mild)	29 (215,872)	51.83 (±15.7)	F: 9 (31.0%)	27.31 (20.61)	5.91 (2.34)
26-50% (moderate)	32 (192,504)	55.66 (±13.03)	F: 13 (40.6%)	26.98 (19.25)	5.91 (2.21)
>50% (severe)	10 (52,502)	44.6 (±16.53)	F: 2 (20.0%)	23.83 (17.27)	8.24 (3.19)

Supplementary Table 8. Quantitative analysis of vascular morphometry based on scored arteriosclerosis in our biopsy cohort (AC_B) and reported hypertension status in four cohorts (AC_B, AC_N, KPMP, HuBMAP). Structures from cases were grouped based on stratified reported scoring and/or hypertension status. Continuous feature distributions are reported as median with interquartile range in brackets while age is reported as mean ± one standard deviation.
F: Female.

Group	Cases (Instances)	Age	Sex	Wall Diameter [µm]	Lumen Diameter [µm]
Arteriosclerosis					
none	129 (Arteries: 2,887 Lumina: 2,720)	45.59 (±15.9)	F: 53 (41.1%)	22.0 (11.78)	28.18 (20.80)
moderate	127 (Arteries: 3,945 Lumina: 3,737)	56.65 (±14.65)	F: 53 (41.7%)	24.02 (12.6)	25.34 (20.77)
severe	33 (Arteries: 1,181 Lumina: 1,042)	55.42 (±14.16)	F: 9 (27.3%)	26.98 (15.33)	22.62 (19.51)
Hypertension					
AC_B					
Hypertension	111 (Arteries: 3,034 Lumina: 2,813)	53.89 (±16.23)	F: 37 (33.3%)	23.83 (12.22)	25.95 (21.12)
Normotension	64 (Arteries: 1,575 Lumina: 1,503)	43.08 (±17.26)	F: 23 (35.9%)	21.94 (11.21)	27.81 (20.43)
AC_N					
Hypertension	24 (Arteries: 7,270 Lumina: 6,857)	61.06 (±17.12)	F: 16 (50.0%)	23.5 (14.74)	26.24 (22.31)
Normotension	6 (Arteries: 1,429 Lumina: 1,379)	57.83 (±19.07)	F: 3 (50.0%)	21.22 (13.77)	27.52 (17.91)
KPMP					
Hypertension	27 (Arteries: 760 Lumina: 675)	30-39: 2<br>40-49: 3<br>50-59: 3<br>60-69: 14<br>70-79: 5	F: 16 (59.3%)	22.61 (13.19)	25.28 (18.52)
Normotension	7 (Arteries: 54 Lumina: 49)	20-29: 1<br>30-39: 2<br>50-59: 2<br>60-69: 2	F: 3 (42.9%)	22.08 (9.59)	28.33 (12.5)
HuBMAP					
Hypertension	5 (Arteries: 762 Lumina: 720)	63.2 (±10.52)	F: 2 (40.0%)	24.39 (14.92)	29.13 (28.9)
Normotension	4 (Arteries: 514 Lumina: 503)	52.75 (±15.13)	F: 2 (50.0%)	20.27 (14.94)	38.4 (28.35)

Supplementary Table 9. Number of cumulative events (i.e., reaching the composite endpoint) of the five fitted univariate Cox proportional hazards models for digital morphometric features in the VALIGA cohort.

Group	n	Year 0	Year 3	Year 6	Year 9	Year 12	Year 15
Tubular Distance [µm]							
< 4.13µm	433	2	11	23	35	41	44
> 4.13µm	211	4	33	57	66	70	71
Tubular Diameter [µm]							
> 24.87µm	546	3	28	55	68	76	79
< 24.87µm	98	3	16	25	33	35	36
Tuft Area [µm²]							
> 6788µm²	462	4	18	38	51	56	57
< 6788µm²	182	2	26	42	50	55	58
Tuft Circularity							
> 0.34	466	1	15	33	44	50	54
< 0.34	175	5	29	47	57	61	61
Tuft Eccentricity							
< 0.66	322	0	11	25	34	36	36
> 0.66	319	6	33	55	67	75	79

Supplementary Table 10. Summary of uni- and multivariate Cox proportional hazards models for digital morphometric features. Multivariate models were adjusted for age (grouped by steps of 10 years), sex (male vs. female), estimated glomerular filtration rate (grouped by steps of 10ml/min/1.73m²) and M, E, S, T as well as C of the Oxford classification for IgA nephropathy. Features were reported as dichotomous based on maximally selected log-rank statistics. Exact p-values of the univariate Cox proportional hazard model were 6.82e-15 for tubular distance, 1.84e-7 for tubular diameter, 4.41e-7 for tuft area, 4.2e-12 for tuft circularity and 2.5e-6 for tuft eccentricity, respectively.
HR: Hazard ratio; CI: Confidence interval, eGFR: estimated glomerular filtration rate; M: Mesangial hypercellularity; E: Endocapillary hypercellularity; S: Segmental sclerosis; T: Tubular atrophy; C: Crescents.

	HR	95% CI	*p*-value	HR	95% CI	*p*-value	HR	95% CI	*p*-value	HR	95% CI	p-value	HR	95% CI	*p*-value
Feature:	Tubular Distance			Tubular Diameter			Tuft Area			Tuft Circularity			Tuft Eccentricity		
Univariate:	4.65	3.16-6.84	<0.0001	2.86	1.93-4.24	<0.0001	2.57	1.78-3.7	<0.0001	3.68	2.55-5.32	<0.0001	2.58	1.74-3.83	<0.0001
Multivariate:															
- Feature	2.03	1.24-3.32	0.0049	1.73	1.12-2.68	0.0137	1.51	1.02-2.25	0.0419	2.04	1.38-3.02	0.0003	1.73	1.15-2.61	0.0091
- Age	0.99	0.85-1.15	0.8943	1.04	0.89-1.21	0.5962	1.04	0.89-1.22	0.5887	1.03	0.88-1.21	0.6928	1.01	0.86-1.17	0.9461
- Sex	1.12	0.71-1.77	0.6345	1.06	0.67-1.67	0.8135	1.01	0.64-1.59	0.973	0.97	0.61-1.53	0.8931	1.01	0.64-1.58	0.981
- eGFR	0.82	0.74-0.92	0.0004	0.82	0.73-0.91	0.0002	0.82	0.74-0.91	0.0003	0.83	0.75-0.93	0.0008	0.81	0.73-0.91	0.0002
- M	1.66	1.13-2.43	0.0101	1.81	1.24-2.66	0.0024	1.81	1.24-2.67	0.0024	1.67	1.13-2.45	0.0096	1.59	1.07-2.34	0.0201
- E	1.08	0.6-1.95	0.8039	1.09	0.6-1.97	0.7786	1.13	0.63-2.06	0.6768	1.12	0.61-2.06	0.7047	1.02	0.56-1.84	0.9589
- S	2.41	1.19-4.88	0.0141	2.36	1.17-4.78	0.017	2.28	1.13-4.6	0.0219	2.18	1.08-4.39	0.0301	2.32	1.15-4.68	0.0189
- T	1.46	1.01-2.1	0.0446	1.62	1.14-2.3	0.0071	1.7	1.22-2.38	0.0019	1.72	1.23-2.42	0.0016	1.77	1.27-2.48	0.0008
- C	1.29	0.74-2.26	0.3732	1.37	0.78-2.42	0.2712	1.34	0.76-2.37	0.3044	1.33	0.76-2.35	0.3222	1.38	0.78-2.42	0.2674

Supplementary Table 11. Summary of the hazard ratios (HR = hazard ratio; exponentiated regression coefficients) and their 2.5-97.5% (95%) confidence intervals from the two fitted multivariate Cox proportional hazards models for digital morphometric features (Digital Biomarkers) and MEST-C histopathology scoring (MEST-C) in the VALIGA cohort. The p-values stem from the individual multivariate analysis for each respective predictive variable. Age, sex and eGFR were not corrected for multiple testing between the two models. Age and estimated glomerular filtration rate were grouped by steps of 10 years and ml/min/1.73m² respectively.
HR: Hazard ratio; CI: Confidence interval. M: Mesangial hypercellularity; E: Endocapillary hypercellularity; S: Segmental sclerosis; T: Tubular atrophy; C: Crescents; eGFR: estimated glomerular filtration rate.

	HR	95% CI	*p*-value
Multivariate:	Digital Biomarkers Model (Feature-based)		
- Tubular Distance	2.01	1.21-3.33	0.0069
- Tubular Diameter	1.37	0.86-2.18	0.181
- Tuft Area	1.14	0.75-1.74	0.5373
- Tuft Circularity	1.79	1.19-2.69	0.0053
- Tuft Eccentricity	1.6	1.04-2.45	0.0325
- Age	0.97	0.83-1.12	0.6625
- Sex	1.04	0.66-1.64	0.8735
- eGFR	0.8	0.73-0.89	0.00004
Multivariate:	MEST-C (Histopathology-based)		
- M	1.74	1.19-2.56	0.0046
- E	1.07	0.59-1.94	0.8159
- S	2.34	1.16-4.73	0.0177
- T	1.81	1.3-2.53	0.0005
- C	1.34	0.76-2.35	0.3144
- Age	1.02	0.88-1.19	0.7881
- Sex	1.02	0.65-1.61	0.9289
- eGFR	0.8	0.72-0.89	0.00001

Supplementary Table 12. Detailed description of clinical and histological data of the multivariate Cox proportional hazards model from 644 patients of the VALIGA cohort. Age and eGFR at time of biopsy are provided as mean ± standard deviation. Categorical features are provided as n with relative frequency in brackets.
F: Female; M: Male; eGFR, estimated glomerular filtration rate, M: Mesangial hypercellularity; E: Endocapillary hypercellularity; S: Segmental sclerosis; T: Tubular atrophy; C: Crescents.

	Overall	Tubular Distance [µm]		Tubular Diameter [µm]		Tuft Area [μm^2]		Tuft Circularity		Tuft Eccentricity	
		> 4.13	< 4.13	< 24.87	> 24.87	< 6788	> 6788	< 0.34	> 0.34	> 0.66	< 0.66
Cases	644	211<br>(32.8%)	433<br>(67.2%)	98<br>(15.2%)	546<br>(84.8%)	182<br>(28.3%)	462<br>(71.7%)	175<br>(27.3%)	466<br>(72.7%)	319<br>(49.8%)	322<br>(50.2%)
Age	36.06<br>(±15.2)	42.4<br>(±14.51)	32.96<br>(±14.58)	36.2<br>(±16.02)	36.03<br>(±15.07)	36.02<br>(±15.12)	36.07<br>(±15.25)	39.6<br>(±14.4)	34.8<br>(±15.26)	37.57<br>(±15.35)	34.66<br>(±14.88)
Sex	F: 181<br>(28.1%)<br>M: 463<br>(71.9%)	F: 40<br>(19.0%)<br>M: 171<br>(81.0%)	F: 141<br>(32.6%)<br>M: 292<br>(67.4%)	F: 25<br>(25.5%)<br>M: 73<br>(74.5%)	F: 156<br>(28.6%)<br>M: 390<br>(71.4%)	F: 53<br>(29.1%)<br>M: 129<br>(70.9%)	F: 128<br>(27.7%)<br>M: 334<br>(72.3%)	F: 45<br>(25.7%)<br>M: 130<br>(74.3%)	F: 134<br>(28.8%)<br>M: 332<br>(71.2%)	F: 90<br>(28.2%)<br>M: 229<br>(71.8%)	F: 89<br>(27.6%)<br>M: 233<br>(72.4%)
eGFR	72.39<br>(±29.95)	51.63<br>(±25.73)	82.51<br>(±26.46)	59.41<br>(±33.77)	74.72<br>(±28.63)	59.09<br>(±30.23)	77.63<br>(±28.2)	56.31<br>(±29.21)	78.29<br>(±27.94)	64.92<br>(±30.96)	79.59<br>(±26.98)
M	M0: 455<br>(70.7%)<br>M1: 189<br>(29.3%)	M0: 130<br>(61.6%)<br>M1: 81<br>(38.4%)	M0: 325<br>(75.1%)<br>M1: 108<br>(24.9%)	M0: 68<br>(69.4%)<br>M1: 30<br>(30.6%)	M0: 387<br>(70.9%)<br>M1: 159<br>(29.1%)	M0: 124<br>(68.1%)<br>M1: 58<br>(31.9%)	M0: 331<br>(71.6%)<br>M1: 131<br>(28.4%)	M0: 111<br>(63.4%)<br>M1: 64<br>(36.6%)	M0: 341<br>(73.2%)<br>M1: 125<br>(26.8%)	M0: 207<br>(64.9%)<br>M1: 112<br>(35.1%)	M0: 245<br>(76.1%)<br>M1: 77<br>(23.9%)
E	E0: 572<br>(88.8%)<br>E1: 72<br>(11.2%)	E0: 193<br>(91.5%)<br>E1: 18<br>(8.5%)	E0: 379<br>(87.5%)<br>E1: 54<br>(12.5%)	E0: 92<br>(93.9%)<br>E1: 6<br>(6.1%)	E0: 480<br>(87.9%)<br>E1: 66<br>(12.1%)	E0: 168<br>(92.3%)<br>E1: 14<br>(7.7%)	E0: 404<br>(87.4%)<br>E1: 58<br>(12.6%)	E0: 153<br>(87.4%)<br>E1: 22<br>(12.6%)	E0: 417<br>(89.5%)<br>E1: 49<br>(10.5%)	E0: 286<br>(89.7%)<br>E1: 33<br>(10.3%)	E0: 284<br>(88.2%)<br>E1: 38<br>(11.8%)
S	S0: 166<br>(25.8%)<br>S1: 478<br>(74.2%)	S0: 36<br>(17.1%)<br>S1: 175<br>(82.9%)	S0: 130<br>(30.0%)<br>S1: 303<br>(70.0%)	S0: 26<br>(26.5%)<br>S1: 72<br>(73.5%)	S0: 140<br>(25.6%)<br>S1: 406<br>(74.4%)	S0: 33<br>(18.1%)<br>S1: 149<br>(81.9%)	S0: 133<br>(28.8%)<br>S1: 329<br>(71.2%)	S0: 23<br>(13.1%)<br>S1: 152<br>(86.9%)	S0: 141<br>(30.3%)<br>S1: 325<br>(69.7%)	S0: 75<br>(23.5%)<br>S1: 244<br>(76.5%)	S0: 89<br>(27.6%)<br>S1: 233<br>(72.4%)
T	T0: 492<br>(76.4%)<br>T1: 130<br>(20.2%)<br>T2: 22<br>(3.4%)	T0: 97<br>(46.0%)<br>T1: 97<br>(44.1%)<br>T2: 21<br>(9.9%)	T0: 395<br>(91.2%)<br>T1: 37<br>(8.6%)<br>T2: 1<br>(0.2%)	T0: 49<br>(50.0%)<br>T1: 37<br>(37.8%)<br>T2: 12<br>(12.2%)	T0: 443<br>(81.1%)<br>T1: 93<br>(17.0%)<br>T2: 10<br>(1.8%)	T0: 104<br>(57.1%)<br>T1: 61<br>(33.5%)<br>T2: 17<br>(9.4%)	T0: 388<br>(84.0%)<br>T1: 69<br>(14.9%)<br>T2: 5<br>(1.1%)	T0: 98<br>(56.0%)<br>T1: 66<br>(37.7%)<br>T2: 11<br>(6.3%)	T0: 391<br>(83.9%)<br>T1: 64<br>(13.7%)<br>T2: 11<br>(2.4%)	T0: 222<br>(69.6%)<br>T1: 80<br>(25.1%)<br>T2: 17<br>(5.3%)	T0: 267<br>(82.9%)<br>T1: 50<br>(15.5%)<br>T2: 5<br>(1.6%)
C	C0: 575<br>(89.3%)<br>C1: 69<br>(10.7%)	C0: 183<br>(86.7%)<br>C1: 28<br>(13.3%)	C0: 392<br>(90.5%)<br>C1: 41<br>(9.5%)	C0: 88<br>(89.8%)<br>C1: 10<br>(10.2%)	C0: 487<br>(89.2%)<br>C1: 59<br>(10.8%)	C0: 163<br>(89.6%)<br>C1: 19<br>(10.4%)	C0: 412<br>(89.2%)<br>C1: 50<br>(10.8%)	C0: 151<br>(86.3%)<br>C1: 24<br>(13.7%)	C0: 421<br>(90.3%)<br>C1: 45<br>(9.7%)	C0: 282<br>(88.4%)<br>C1: 37<br>(11.6%)	C0: 290<br>(90.1%)<br>C1: 32<br>(9.9%)

Supplementary Table 13. Data availability for internal and external cohorts (n plus relative frequency in brackets). Histopathology includes the scored amount of interstitial fibrosis and tubular atrophy (IFTA) and the MEST-C-score for each biopsy (in case of VALIGA).
eGFR: estimated glomerular filtration rate.

Cohort	Cases [n]	Age	Sex	Histo-pathology	eGFR	Proteinuria	Hypertension
AC_B	320	320 (100%)	320 (100%)	320 (100%)	236 (74%)	122 (38%)	283 (88%)
AC_N	30	30 (100%)	30 (100%)	0 (0%)	0 (0%)	0 (0%)	30 (100%)
KPMP	36	36 (100%)	36 (100%)	0 (0%)	34 (94%)	19 (53%)	34 (94%)
HuBMAP	9	9 (100%)	9 (100%)	0 (0%)	0 (0%)	0 (0%)	9 (100%)
VALIGA	648	648 (100%)	648 (100%)	648 (100%)	644 (99%)	621 (96%)	0 (0%)

Supplementary Table 14. Diagnoses present in the AC_B cohort based on the Banff-Classification for transplant pathology[1] and the Mayo-Classification for glomerulonephritides[2]. For cases with multiple diagnoses the amount of overlapping cases was further reported.
ABMR: Antibody mediated rejection; TCMR: T-cell mediated rejection; GN: Glomerulonephritis; GBM: Glomerular basement membrane; FSGS: Focal segmental glomerulosclerosis. IgAN: IgA nephropathy, DN: Diabetic nephropathy, HTN: Hypertensive nephropathy, MPGN: Membranoproliferative glomerulonephritis, MPO: Myeloperoxidase, PR3: Proteinase 3, C3: Complement component 3.

Class	Category	Diagnosis	Cases [n]	Overlap
Transplant	Banff Category 1	normal	32	-
	Banff Category 2	ABMR	7	-
	Banff Category 3	Borderline TCMR	13	-
	Banff Category 4	TCMR	9	-
	Banff Category 5	Other (e.g., Polyomavirus nephritis)	41	-
	Mixed	combined category 2 & 3/4 rejection	6	-
Native	Immune-complex GN	IgA nephropathy (IgAN)	47	3 (DN/HTN)
		Lupus nephritis (LN)	13	1 (Memb. GN)
		Membranous GN (Memb. GN)	9	1 (LN)
		Membranoproliferative GN (MPGN)	4	1 (Interst. Neph.)
	Pauci-immune GN	MPO/PR3 ANCA GN (Pauci)	31	1 (Interst. Neph.)
	C3 glomerulopathy	C3 glomerulopathy	3	-
	Anti-GBM GN	Anti-GBM GN	1	-
	Podocytopathy	Minimal change disease (MCD)	16	-
		primary FSGS	1	-
	Tubulointerstitial	Interstitial nephritis (Interst. Neph)	11	2 (Pauci/MPGN)
		Cast nephropathy	4	-
		Acute tubular injury (isolated)	5	-
		Light-chain proximal tubulopathy	1	-
	Vascular	Hypertensive nephropathy (HTN)	9	3 (IgAN/DN)
		Thrombotic microangiopathy	4	-
	Other	Diabetic nephropathy (DN)	8	3 (IgAN/HTN)
		Amyloidosis	7	-
		IgM nephropathy	1	-
	Normal	age-appropriate kidney biopsy	17	-
	Non-specific	descriptive biopsy report	27	-

Supplementary Table 15. Number of instances that were annotated as ground truth in the development (training and internal validation) and testing/external validation of the structure segmentation convolutional neural network. Instances which were present in multiple patches were only counted once.

No. of Instances						
**Cohort**	Tubule	Glomerulus	Glomerular Tuft	Non-Tissue Background	Artery	Arterial Lumen
AC_B	14,492	761	627	605	1,887	2,041
AC_N	3,246	186	194	55	189	202
KPMP	1,070	50	46	38	177	189
HuBMAP	924	62	58	23	73	92
∑	19,732	1,059	925	721	2,326	2,524

Supplementary Table 16. Overview of the annotated ground truth data for the development, testing and external validation of the tissue segmentation and structure segmentation convolutional neural network. Our internal cohorts AC_B & AC_N for CNN development were split into training, internal validation during training, and testing for performance evaluation. Unseen whole-slide images from the two external cohorts (KPMP & HuBMAP) were used for external validation.
WSI: Whole-slide image.

	Tissue Segmentation				Structure Segmentation							
	Number of WSIs				Number of WSIs				Number of Patches			
Cohort	Development		Testing/ external Validation	Σ	Development		Testing/ external Validation	Σ	Development		Testing/ external Validation	Σ
	Training	Internal Validation			Training	Internal Validation			Training	Internal Validation		
AC_B	597	149	191	937	54	2	12	68	2,621	78	463	3,162
AC_N	19	5	6	30	10	2	5	17	200	30	201	431
KPMP	0	0	80	80	0	0	5	5	0	0	240	240
HuBMAP	0	0	9	9	0	0	5	5	0	0	198	198
Σ	616	154	286	1,056	64	4	27	95	2,821	108	1,210	4,031

Supplementary Table 17. Overview of cases and their respective diagnoses on which annotations were performed. The number of cases where diagnoses overlap (i.e., simultaneous presence of two or more kidney diseases) is provided in brackets. Diagnoses only overlap in the specific training or test set. Non-specific cases mostly were biopsies with considerable interstitial fibrosis and glomerulosclerosis. In the external KPMP cohort only the presence of either acute kidney injury or chronic kidney disease was reported.
ABMR: Antibody mediated rejection; TCMR: T-cell mediated rejection; GN: Glomerulonephritis; C3: Complement component 3; AKI: Acute kidney injury; CKD: Chronic kidney disease.

Cohort	Diagnosis	Cases Training (n)	Cases Test/Validation (n)
AC_B	ABMR	2	2 (1)
	TCMR	2	1 (1)
	Polyomavirus nephritis	1	-
	Immune-complex GN (not further differentiated)	1	-
	IgA nephropathy	18 (2)	3 (1)
	Lupus nephritis	5	2
	Membranous GN	-	1
	Membranoproliferative GN	1	-
	Pauci-immune GN	10	1
	C3 glomerulopathy	1	-
	Minimal change disease	2	-
	Hypertensive nephropathy	1 (1)	2 (2)
	Thrombotic microangiopathy	3	-
	Diabetic nephropathy	6 (3)	2 (2)
	Amyloidosis	1	-
	Non-specific (chronic damage)	5	2
AC_N	Transplant nephrectomy	-	2
	Tumour nephrectomy	9	3
KPMP (external validation)	AKI	-	1
	CKD	-	4
HuBMAP (external validation)	Healthy nephrectomy	-	5

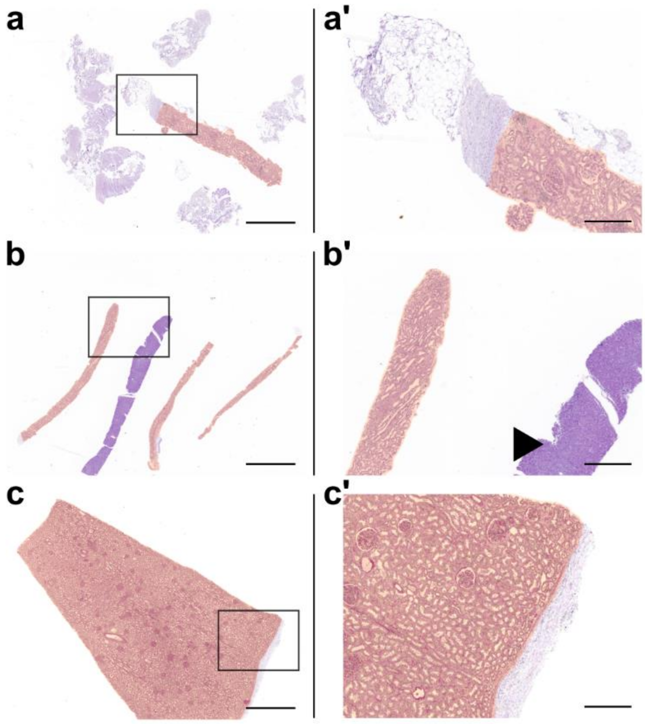

Supplementary Figure 1. Representative whole-slide images with prediction overlay (orange) of the tissue segmentation convolutional neural network. Kidney tissue from nephrectomy and biopsy specimens was precisely segmented. Non-kidney tissue of various origins such as connective tissue (a, a'), fat (a, a'), muscle (a, a'), fibrous capsule (c, c') and even liver tissue (b, arrowhead b') were not recognized as kidney tissue and therefore excluded. Left (a-c) scale bar: 2mm; right (a'-c') scale bar: 0.5mm.

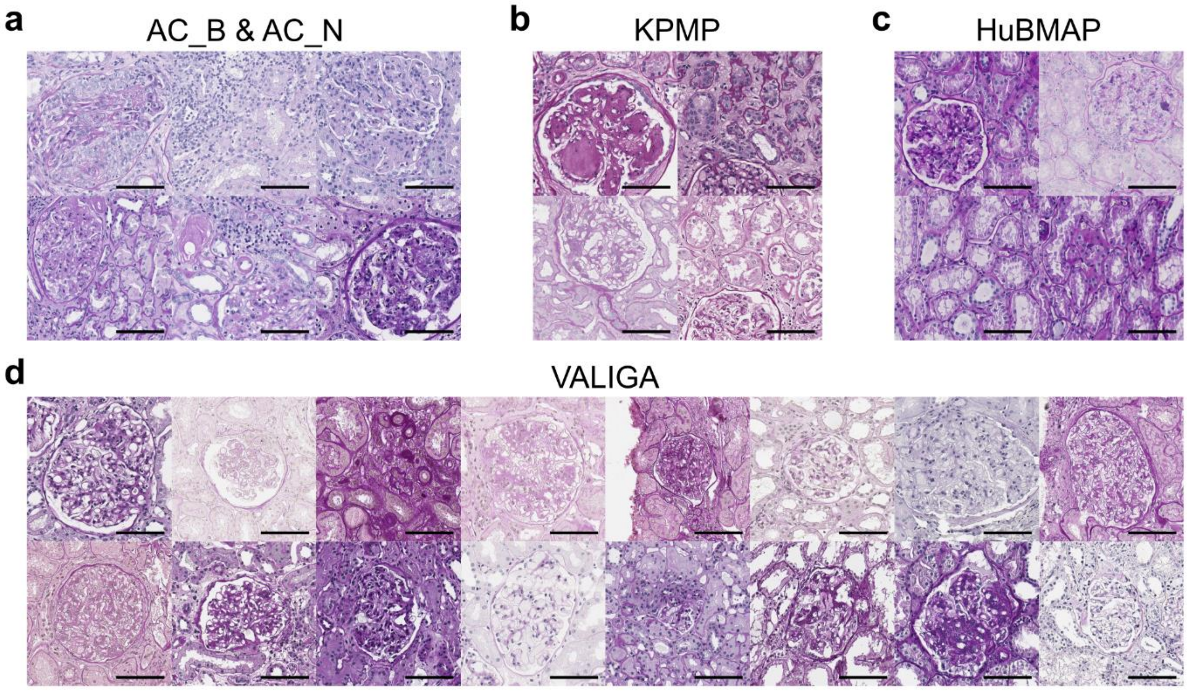

Supplementary Figure 2. Variation in Periodic Acid Schiff-staining between the five included cohorts (a-d). Internal cohorts (a) are combined due to similar staining and cutting routines. Scale bar size is 100µm.

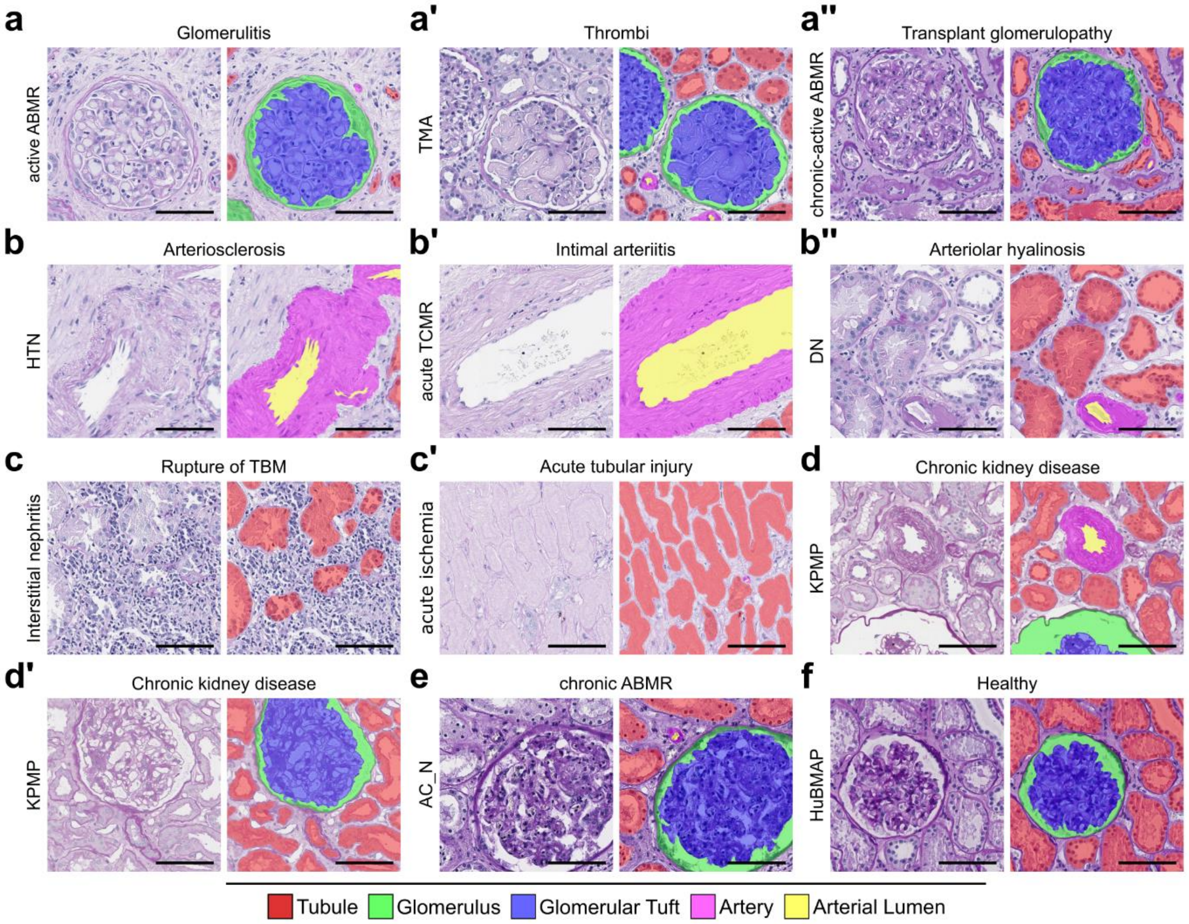

Supplementary Figure 3. Additional representative segmentation visualisations for glomeruli (a-a''), arteries (b-b'') and tubules (c-c') as well as segmentations from the internal nephrectomy cohort (e) and external cohorts (d-d', f). For biopsies from the KPMP cohort (d-d') only chronic kidney disease or acute kidney injury were reported as diagnoses. Despite major differences in cutting and staining protocols, histological structures were precisely segmented. Scale bar size is 100µm.

ABMR: Antibody-mediated rejection; TMA: Thrombotic microangiopathy; HTN: Hypertensive nephropathy; TCMR: T-cell mediated rejection; DN: Diabetic nephropathy; TBM: Tubular basement membrane.

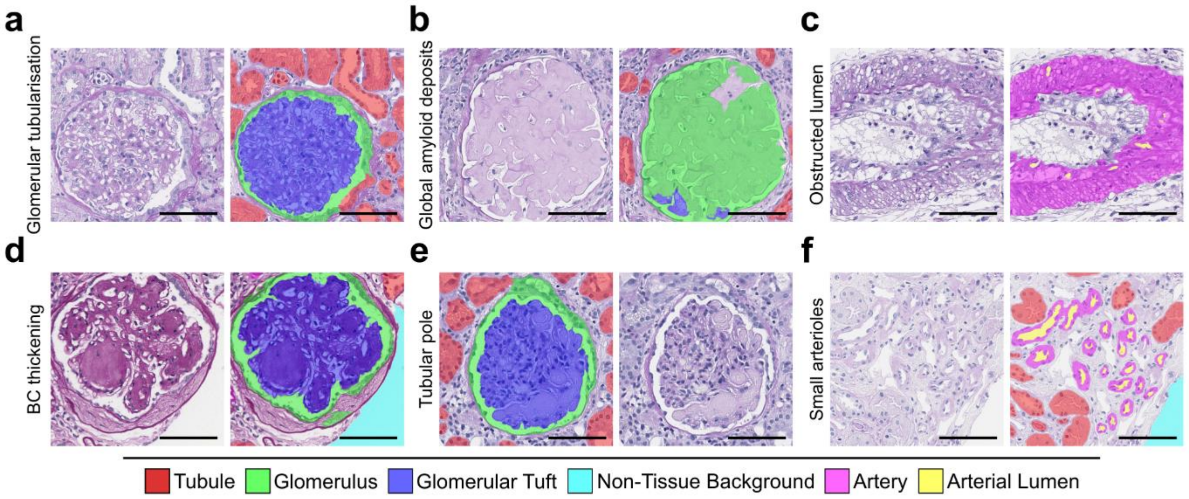

Supplementary Figure 4. Representative selection of imprecise segmentation results from the AC_B (a-b, e-f), AC_N (c) and KPMP (d) cohort of glomeruli, tubules and arteries. Scale bar size is 100µm.
BC: Bowman's capsule.

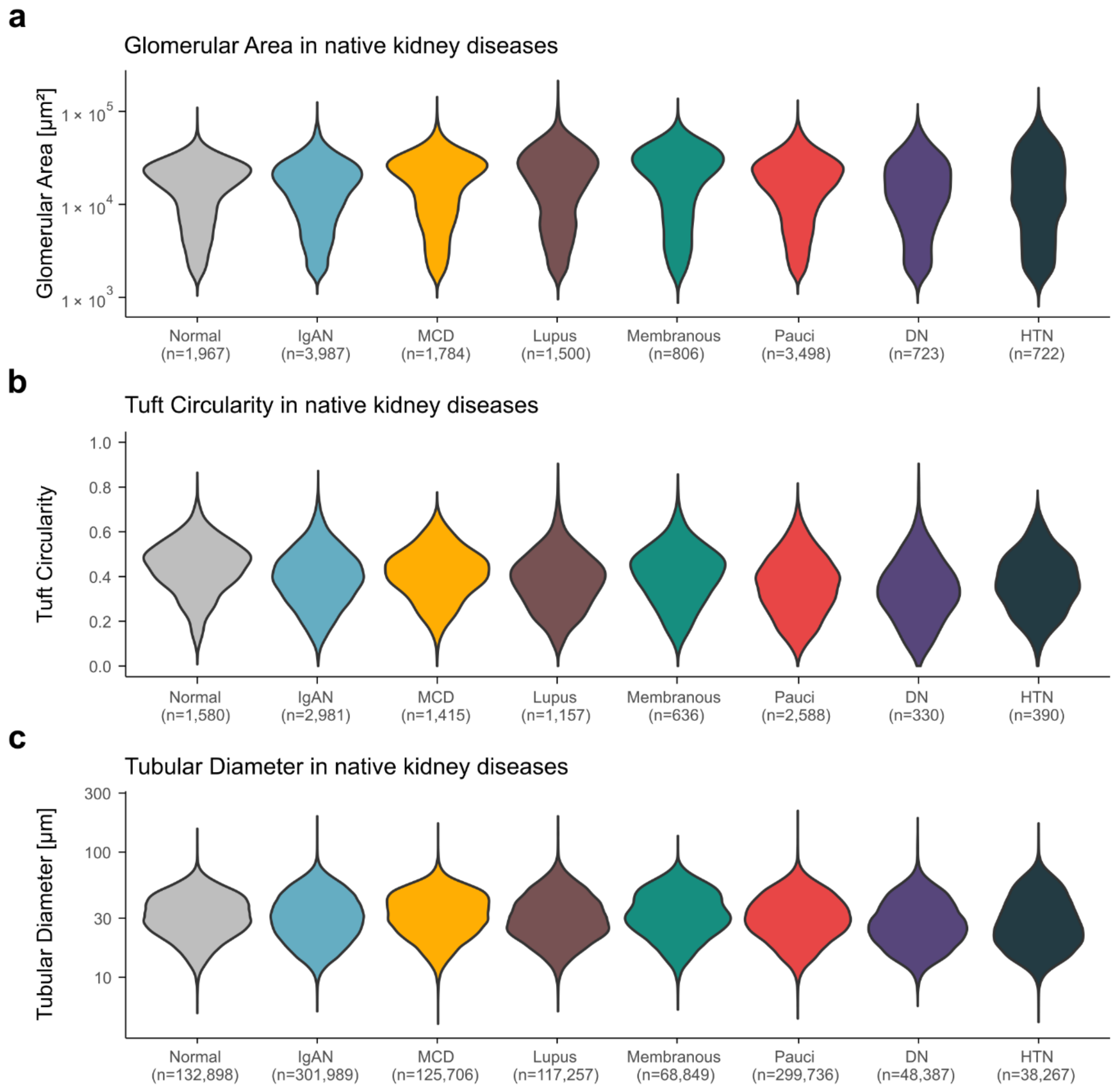

Supplementary Figure 5. Next-generation morphometry analysis for disease comparison. Analysis of (a) 14,988 glomeruli, (b) 11,077 glomerular tufts and (c) 1,133,089 tubules on instance-level based on the reported diagnosis in the internal AC_B cohort. Different diseases display unique distributions for different features underlining the complex influence of native kidney diseases on its histomorphology. Source data are provided as a Source Data file.
IgAN: IgA nephropathy; MCD: Minimal change disease; Lupus: lupus nephritis; Membranous: Membranous glomerulonephritis; Pauci: Pauci-immune glomerulonephritis, DN: Diabetic nephropathy; HTN: Hypertensive nephropathy.

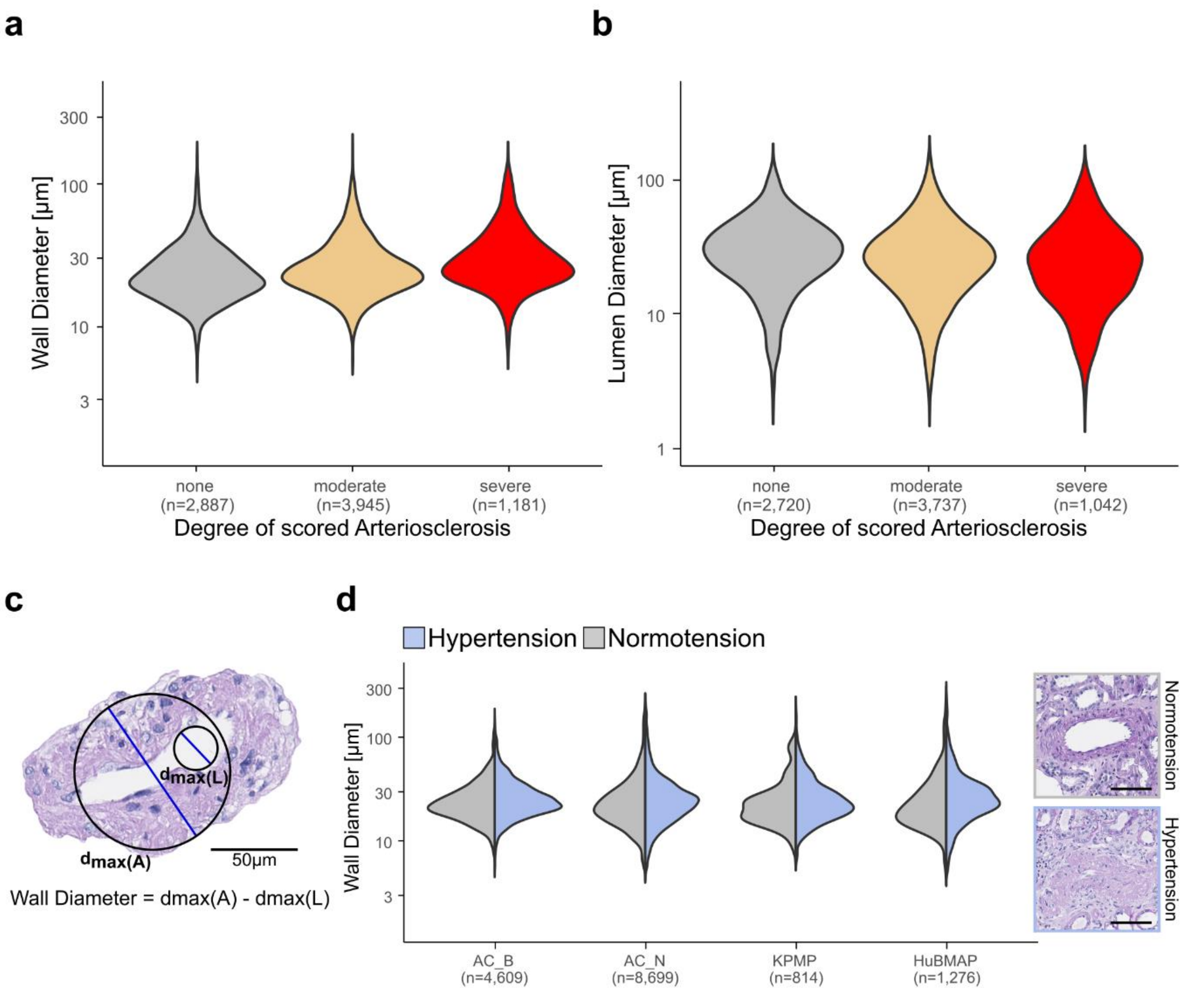

Supplementary Figure 6. Analysis of (a) artery wall and (b) lumen diameter based on the degree of arteriosclerosis scored by two nephropathologists. Wall and lumen diameters were assessed on instance-level. The reported degree of arteriosclerosis was assigned as a global label to all arteries and arterioles present in the whole-slide images of the associated biopsy. For the analysis of lumen diameter arteries without a lumen were excluded. (c) Feature visualisation for arterial/arteriolar wall diameter. (d) Analysis of the wall diameter on instance-level based on the presence of hypertension regardless of aetiology in two internal and two external cohorts where hypertension status was reported. Scale bar size (D) is 100µm. Source data are provided as a Source Data file.

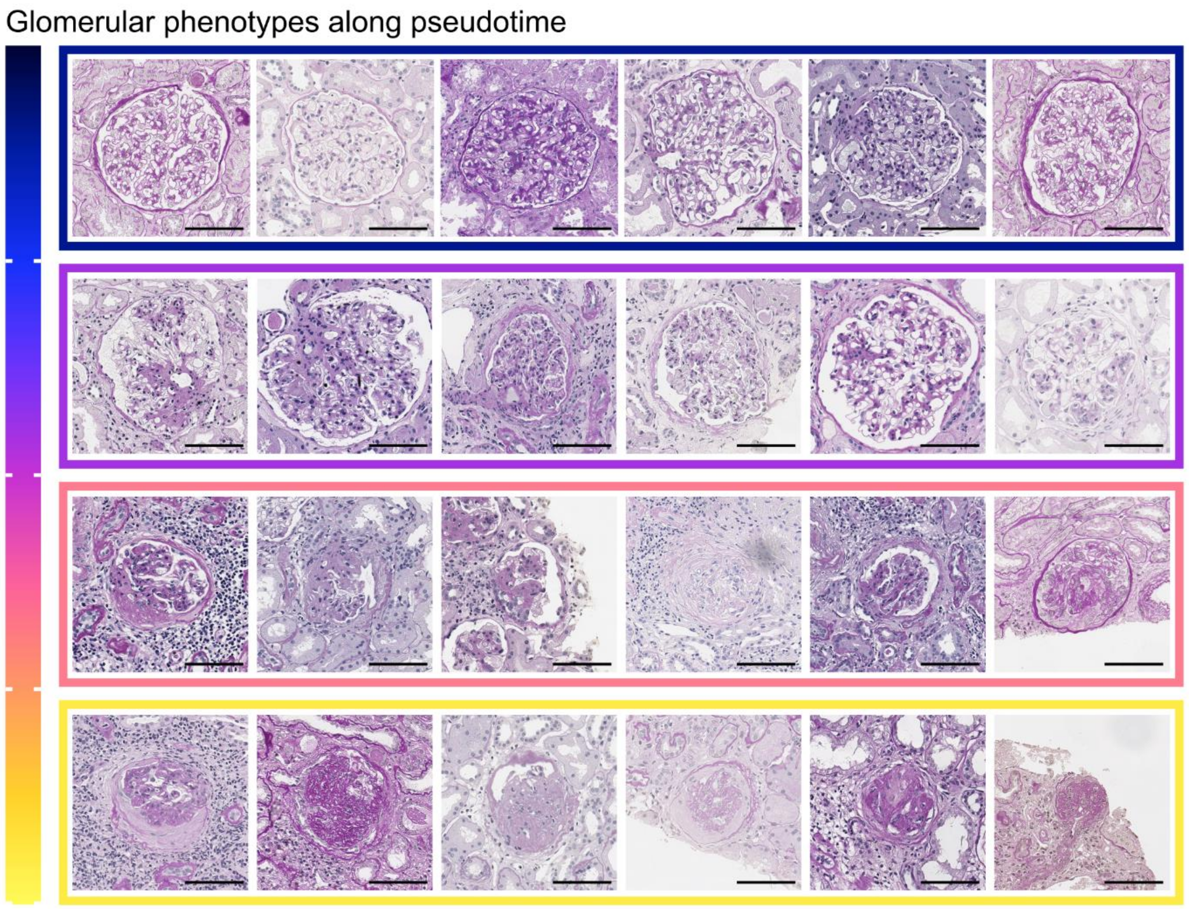

Supplementary Figure 7. Additional examples of glomerular phenotypes from the pseudotime trajectory analysis (blue - healthy phenotype to yellow - very diseased phenotype) of the VALIGA trial. Colouring of borders displays the relative position of the displayed structures along the trajectory. Scale bar size is 100µm.

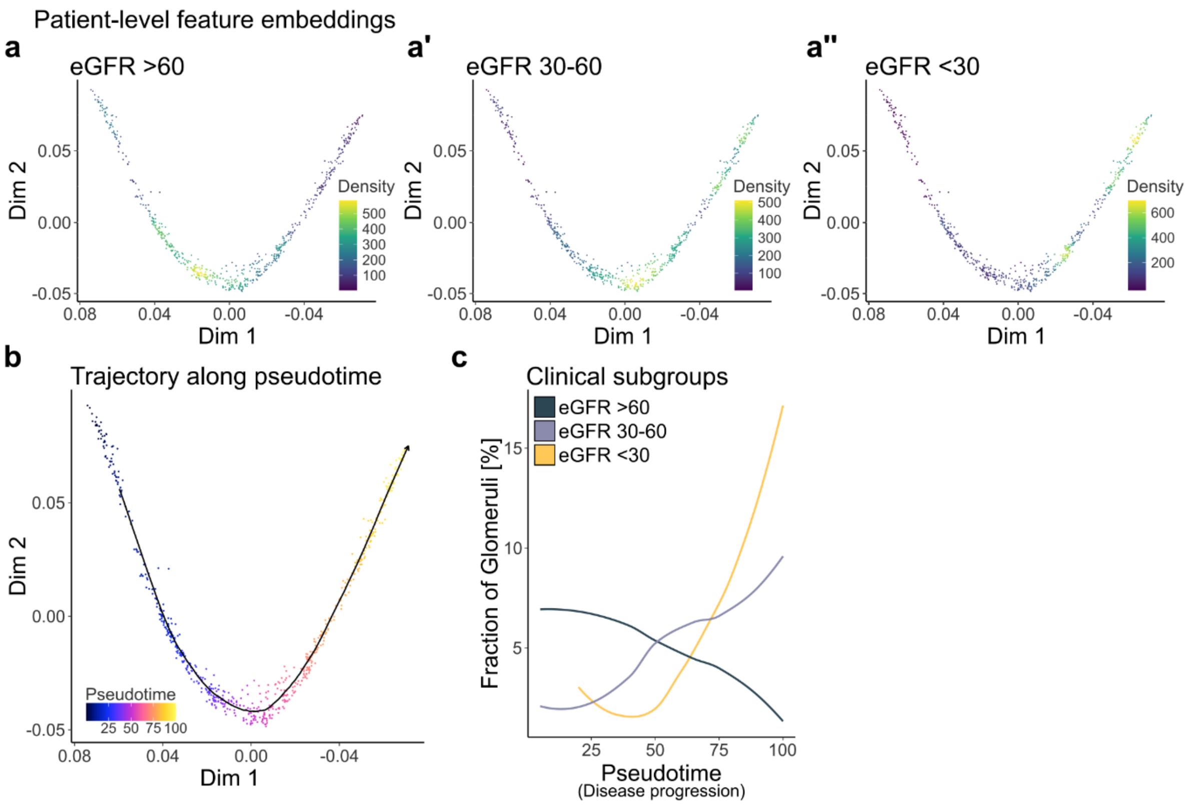

Supplementary Figure 8. Pseudotime analysis of NGM-derived glomerular and tubular features aggregated on patient-level identifies patients along a disease progression trajectory in IgA nephropathy (IgAN). (a-a'') Diffusion map embedding of 634 patients from the VALIGA trial with IgAN based on the reported estimated glomerular filtration rate (eGFR) [ml/min/1.73m²]. (b) Diffusion mapping of patients with pseudotime indicating ordering of patients along their progression from healthy to diseased. (c) Morphometric progression of patients in clinical subgroups based on the overall reported eGFR. Source data are provided as a Source Data file.
eGFR: estimated glomerular filtration rate; Dim: Diffusion map.

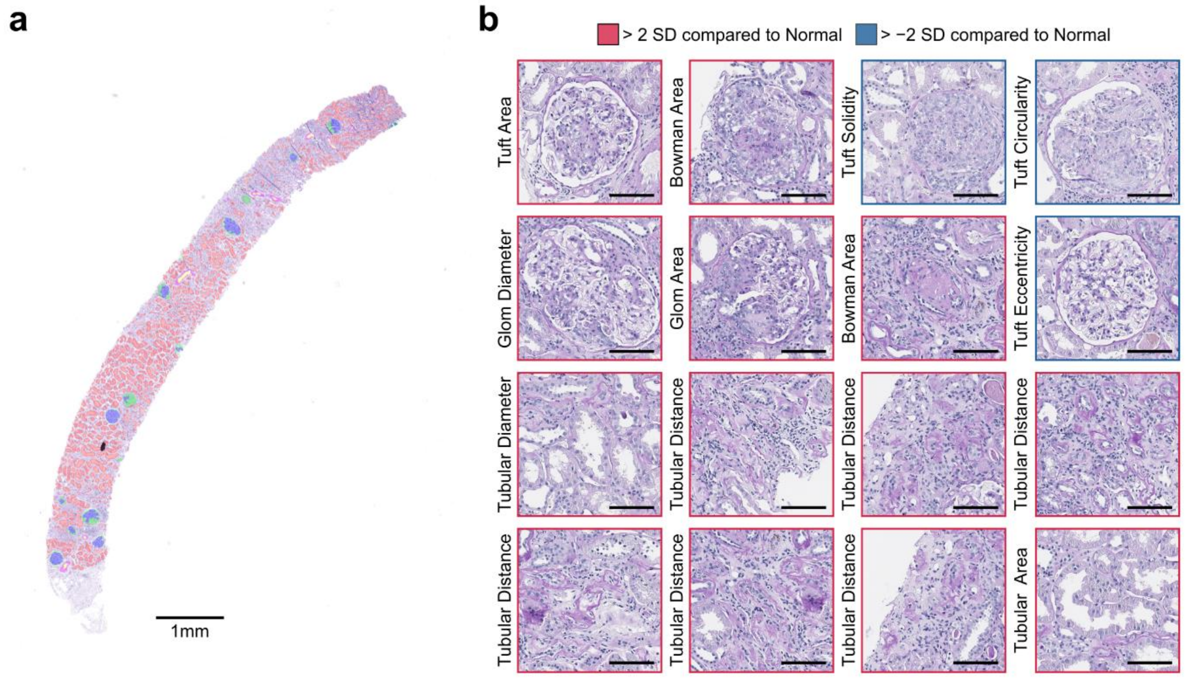

Supplementary Figure 9. Whole-slide segmentation and automated visualisation of feature outliers. (a) Segmentation of a representative IgA nephropathy case from the *AC_B* cohort. (b) Visualisation of patches which display at centre a glomerulus, glomerular tuft or tubule including additional context. Structures in the centre of the patch morphometrically diverge two standard deviations (SD) above (red) or below (blue) the feature specific distribution in normal biopsies. Scale bar size (B) is 100µm.

Supplementary References:

1. Roufosse, C. *et al.* A 2018 Reference Guide to the Banff Classification of Renal Allograft Pathology. *Transplantation* **102**, 1795–1814 (2018).
2. Sethi, S. *et al.* Mayo Clinic/Renal Pathology Society Consensus Report on Pathologic Classification, Diagnosis, and Reporting of GN. *J. Am. Soc. Nephrol.* **27**, 1278–1287 (2016).
