# Supplementary material for: Next-Generation Morphometry for pathomics-data mining in histopathology
Source: Nat Commun. 2023 Jan 28;14:470. doi: 10.1038/s41467-023-36173-0 (PMC9884209; doi:10.1038/s41467-023-36173-0)
Supplement: Supplementary file 3 — Reporting Summary [file 41467_2023_36173_MOESM3_ESM.pdf]

nature portfolio

Corresponding author(s): Prof. Dr. P. Boor

Last updated by author(s): Dec 19, 2022

Reporting Summary

Nature Portfolio wishes to improve the reproducibility of the work that we publish. This form provides structure for consistency and transparency in reporting. For further information on Nature Portfolio policies, see our Editorial Policies and the Editorial Policy Checklist.

Statistics

For all statistical analyses, confirm that the following items are present in the figure legend, table legend, main text, or Methods section.

n/a	Confirmed	
☐	☒	The exact sample size (*n*) for each experimental group/condition, given as a discrete number and unit of measurement
☐	☒	A statement on whether measurements were taken from distinct samples or whether the same sample was measured repeatedly
☐	☒	The statistical test(s) used AND whether they are one- or two-sided<br>*Only common tests should be described solely by name; describe more complex techniques in the Methods section.*
☐	☒	A description of all covariates tested
☐	☒	A description of any assumptions or corrections, such as tests of normality and adjustment for multiple comparisons
☐	☒	A full description of the statistical parameters including central tendency (e.g. means) or other basic estimates (e.g. regression coefficient) AND variation (e.g. standard deviation) or associated estimates of uncertainty (e.g. confidence intervals)
☐	☒	For null hypothesis testing, the test statistic (e.g. F, t, r) with confidence intervals, effect sizes, degrees of freedom and P value noted<br>*Give P values as exact values whenever suitable.*
☒	☐	For Bayesian analysis, information on the choice of priors and Markov chain Monte Carlo settings
☒	☐	For hierarchical and complex designs, identification of the appropriate level for tests and full reporting of outcomes
☒	☐	Estimates of effect sizes (e.g. Cohen's *d*, Pearson's *r*), indicating how they were calculated

Our web collection on statistics for biologists contains articles on many of the points above.

Software and code

Policy information about availability of computer code

Data collection	The freeware QuPath v0.2.3 was used for annotation of whole-slide images.
Data analysis	The code for the segmentation models in this study is openly accessible at: git-ce.rwth-aachen.de/labooratory-ai/flash. Morphometry data aggregation and statistical analysis was performed within the R enviroment version v4.0.3 using the packages tidyverse v1.3.1, dplyr v1.0.8, gdata v2.18.0, broom v0.7.12, kSamples v1.2-9, PMCMRplus v1.9.3, simpleboot v1.1-7, boot v1.3-28, survival v3.3-0, survminer v0.4.9, maxstat v0.7-25, ggplot2 v3.3.3 and cowplot v1.1.1.

For manuscripts utilizing custom algorithms or software that are central to the research but not yet described in published literature, software must be made available to editors and reviewers. We strongly encourage code deposition in a community repository (e.g. GitHub). See the Nature Portfolio guidelines for submitting code & software for further information.

Data

Policy information about availability of data

All manuscripts must include a data availability statement. This statement should provide the following information, where applicable:

- Accession codes, unique identifiers, or web links for publicly available datasets
- A description of any restrictions on data availability
- For clinical datasets or third party data, please ensure that the statement adheres to our policy

Data availability statement

The pathomics data, associated clinical data and many segmentation images (>2000 paired image patches (PAS plus segmentation)) generated in this study have been deposited in our github repository: https://git-ce.rwth-aachen.de/labooratory-ai/flash. The raw whole slide image data are available under restricted access for privacy protection reasons, access can be obtained by directly contacting Peter Boor, Institute of Pathology, RWTH Aachen University Clinic, Aachen, Germany, pboor@ukaachen.de (for the AC_B and AC_N datasets) or Rosanna Coppo, Fondazione Ricerca Molinette, Torino, Italy, rosanna.coppo@unito.it (for the VALIGA dataset). In general, the requests will be evaluated within 4 weeks based on institutional and trial policies. Data can only be shared for non-commercial research purposes and requires a data transfer agreement.
The aggregated data and raw data used to create figure panels generated in this study are provided in the Supplementary Information/Source Data files. The public external image and clinical data used in this study are available in the KPMP (atlas.kpmp.org/repository) and HubMAP (portal.hubmapconsortium.org) databases.

Code availability statement

The source code for FLASH and instructions on how to use it are are freely available at: git-ce.rwth-aachen.de/labooratory-ai/flash.

Human research participants

Policy information about studies involving human research participants and Sex and Gender in Research.

Reporting on sex and gender

All clinical data in our study only contains information regarding the sex of the patients as gathered within the pathology information system and we do not refer to the patients gender in the manuscript. We have provided clinical data regarding the distribution of sex in the different cohorts and experiments in the supplementary information.

Population characteristics

Two internal, single centre (Aachen Biopsy & Aachen Nephrectomy, AC_B & AC_N), and three external, multi-centre cohorts (HubMAP, KPMP, VALIGA) of kidney biopsies and nephrectomies were included. The two largest cohorts in this study are AC_B and VALIGA, covering approximately 92% of total cases used. Demographic and clinical characteristics between cohorts were comparable, apart from younger patients and more males in the VALIGA cohort, as well as reduced kidney function assessed by estimated glomerular filtration rate (eGFR), which was more common in the AC_B cohort and a higher prevalence of hypertension in the AC_N cohort.

Recruitment

For development, validation and application of FLASH, whole-slide images (WSIs) and clinical data from five cohorts were gathered: two internal, i.e. in-house cohorts from the Institute of Pathology in Aachen, for development, i.e. Aachen Biopsy (AC_B) and Nephrectomy (AC_N), and three external cohorts from other centers, i.e., Kidney Precision Medicine Project (KPMP, NCT04334707), the Human BioMolecular Atlas Program (HuBMAP) and the European Validation Study of the Oxford Classification of IgA nephropathy (VALIGA) trial.
Aachen Biopsy cohort (AC_B). A database search identified 355 kidney biopsy cases in the archive of the Institute of Pathology of the RWTH Aachen university clinic within the inclusion period (January 1st 2017 - May 1st 2021).
Aachen Nephrectomy cohort (AC_N). 30 nephrectomy specimens (inclusion period: 2013 - 2021) were included.
Kidney Precision Medicine Project (KPMP). Data from the KPMP repository was accessed on 15th March 2021 and consists of 90 PAS-stained WSIs from patients with either acute kidney injury (AKI), chronic kidney disease (CKD) or healthy tumour nephrectomies.
Human BioMolecular Atlas Program (HubMAP). The HuBMAP database (accessed on 15th March 2021) contains 22 nephrectomy specimens from 12 deceased organ donors. 13 cryo- sections were excluded since they were out of distribution (we only trained on FFPE material), with the final cohort consisting of nine nephrectomy WSIs from nine cases.
European Validation Study of the Oxford Classification of IgA nephropathy (VALIGA). From the initial VALIGA trial cohort, 768 cases could be identified and digitised (scanned). Overall, 106 cases were excluded. An additional 14 cases were excluded on slide level due to artefacts, with in total, 648 PAS-stained WSIs of 648 cases being included.
Patients from the five cohorts were recruited retrospectively and refined based on defined exclusion criteria (Life sciences study design: Data exclusions). Exclusion criteria were solely based on ensuring high quality of the kidney specimen and histological slides.

Ethics oversight

Data collection and analysis in this study was performed in accordance with the Declaration of Helsinki and was approved by the local ethics committee of the RWTH Aachen University (EK-No. 315/19). All analyses were performed retrospectively in an anonymous fashion and the need for informed consent was waived by the local ethics and privacy committee for all datasets.

Note that full information on the approval of the study protocol must also be provided in the manuscript.

Field-specific reporting

Please select the one below that is the best fit for your research. If you are not sure, read the appropriate sections before making your selection.

[x] Life sciences [ ] Behavioural & social sciences [ ] Ecological, evolutionary & environmental sciences

For a reference copy of the document with all sections, see nature.com/documents/nr-reporting-summary-flat.pdf

Life sciences study design

All studies must disclose on these points even when the disclosure is negative.

Sample size	Sample sizes in the respective cohorts were determined by data availability. Annotations for development of the tissue segmentation model were performed until performance could not be further improved substantially. Annotations for development of the structure segmentation model were performed and performance was assessed in multiple cycles until satisfying performance was reached.
Data exclusions	Following exclusion criteria were used in all cohorts: i) no kidney tissue in the specimen, ii) no Periodic Acid Schiff-stained slide available, iii) only cryosections available, iv) specimen containing less than eight glomeruli, unless a definitive pathological diagnosis could be made, v) large artefacts present on the slide, vi) insufficient scan quality (e.g., major part of tissue being out of focus and blurred), vii) insufficient stain quality (e.g., unstained tissue) and viii) broken slides.
Replication	CNN-based segmentation and feature extraction were fully reproducible. We provide Source Data files for the replication of each plot provided in the manuscript's figures.
Randomization	Splits for training, internal validation and testing were performed randomised on case-level.
Blinding	Investigators were not blinded during the annotation process since we specifically aimed at including a diverse spectrum of morphology and kidney diseases in the datasets. Randomisation was performed blinded.

Reporting for specific materials, systems and methods

We require information from authors about some types of materials, experimental systems and methods used in many studies. Here, indicate whether each material, system or method listed is relevant to your study. If you are not sure if a list item applies to your research, read the appropriate section before selecting a response.

Materials & experimental systems

n/a	Involved in the study	
☒	☐	Antibodies
☒	☐	Eukaryotic cell lines
☒	☐	Palaeontology and archaeology
☒	☐	Animals and other organisms
☐	☒	Clinical data
☒	☐	Dual use research of concern

Methods

n/a	Involved in the study	
☒	☐	ChIP-seq
☒	☐	Flow cytometry
☒	☐	MRI-based neuroimaging

Clinical data

Policy information about clinical studies

All manuscripts should comply with the ICMJEguidelines for publication of clinical research and a completedCONSORT checklist must be included with all submissions.

Clinical trial registration	n/a
Study protocol	This study was not performed as a clinical trial, but as a retrospective analysis of clinical data.
Data collection	Internal cohorts were assembled based on the defined inclusion periods (2017-2021 for biopsy specimen and 2013-2021 for nephrectomy specimen). All slides and additional clinical data from external cohorts were included in this study which were available at the time of access.
Outcomes	Outcome of disease progression in IgA nephropathy cases from the VALIGA cohort was defined as reaching end-stage kidney disease and/or halving of initial estimated glomerular filtration rate at the time of biopsy within 15 years.
